# Synthetic closed-loop gene circuit for phenylalanine regulation

**DOI:** 10.1093/nar/gkaf1151

**Published:** 2025-11-04

**Authors:** Silvia Galvan, Yu-Qing Xie, Ana P Teixeira, Martin Fussenegger

**Affiliations:** Department of Biosystems Science and Engineering, ETH Zurich, Klingelbergstrasse 48, Basel CH-4056, Switzerland; Department of Biosystems Science and Engineering, ETH Zurich, Klingelbergstrasse 48, Basel CH-4056, Switzerland; Department of Biosystems Science and Engineering, ETH Zurich, Klingelbergstrasse 48, Basel CH-4056, Switzerland; Department of Biosystems Science and Engineering, ETH Zurich, Klingelbergstrasse 48, Basel CH-4056, Switzerland; Faculty of Science, University of Basel, Klingelbergstrasse 48, Basel CH-4056, Switzerland

## Abstract

Genetic programming of cells offers significant potential for developing next-generation cell-based therapies that detect and respond to signals within the patient’s body to treat chronic diseases. Closed-loop systems designed to self-regulate in response to abnormal biomarker levels are particularly attractive for biomedical applications. In this study, we engineered PRO (phenylalanine regulation orchestration), a cell-autonomous genetic system capable of sensing and degrading elevated levels of phenylalanine, an essential amino acid that accumulates to pathological levels in patients with phenylketonuria (PKU). Central to this system is a transcriptional switch relying on the regulatory domain of human phenylalanine hydroxylase, which dimerizes in the presence of phenylalanine to reconstitute a split transcription factor, thereby inducing gene expression from a synthetic promoter. This sensing module was optimized through random mutagenesis to ensure responsiveness to high concentrations of phenylalanine, enabling robust and dose-dependent activation of protein expression. Engineered human PRO cells containing the sensor driving the expression of phenylalanine-degrading enzymes effectively detected and degraded excess phenylalanine. Microencapsulated PRO cells efficiently reduced phenylalanine levels to the normal physiological range when cultured in human whole blood. Finally, as proof of concept, we showed that alginate-encapsulated PRO cells intraperitoneally implanted in a PKU mouse model significantly lowered blood phenylalanine levels compared to controls implanted with non-engineered cells. Our results suggest that synthetic self-regulating systems are promising candidates for the treatment of metabolic diseases characterized by accumulation of toxic metabolites.

## Graphical Abstract

**Graphical Abstract:**
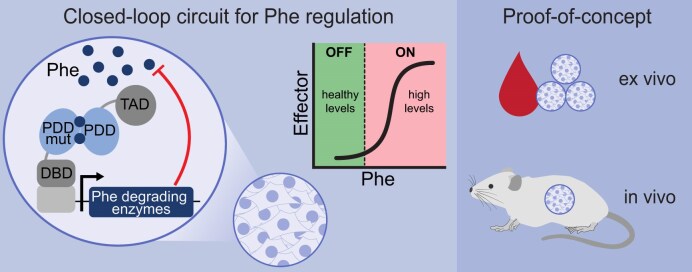

## Introduction

Engineering transcriptional systems that respond to desired inputs have enabled the development of designer cells with programmable and tunable therapeutic outputs. To perform computer-like functions and process complex information, designer cells are typically engineered with a sensor module to detect specific inputs and an effector module, which processes and translates the information into the desired output. In recent years, much effort has focused on designing gene switches that enable user-defined phenotypic control (open-loop circuits) or cell-autonomous phenotypic control (closed-loop circuits). In the first case, exogenously administered molecular [1] or physical [2] inputs are applied to activate the designer cells. Instead, the latter approach is particularly attractive when applied to metabolic disorders, which often require lifelong daily drug administration or lack effective treatments. In such cases, gene–network devices, integrated into designer cells and functionally connected to self-regulating mechanisms, could sense and manage metabolic imbalances by autonomously controlling the therapeutic program. This would require engineering robust genetic circuits that activate in response to elevated levels of biomarker or dysregulated metabolic compounds, while remaining inactive under normal physiological conditions. Examples of closed-loop systems include the regulation of glucose homeostasis [3], the stabilization of elevated insulin levels [4], reduction of dysregulated uric acid levels [5], and the detection and elimination of bacterial infections [6]. However, to our knowledge, no cell-autonomous systems have yet been developed to manage abnormal levels of essential metabolites, such as amino acids, that are characteristic of certain hereditary metabolic disorders [7–9].

Here, we report a synthetic mammalian self-regulation system designed to dynamically respond to l-phenylalanine (Phe), an essential amino acid that accumulates to toxic levels in patients with phenylketonuria (PKU) due to mutations in the Phe hydroxylase (PAH) gene, leading to severe neurotoxicity [10–13]. According to European guidelines, lifelong treatment is recommended for adult PKU patients with Phe levels exceeding 600 µM with target levels being in the range of 120–600 µM [14, 15]. On the other hand, Phe deficiency can also cause serious complications, as Phe is essential for protein synthesis and serves as a precursor of neurotransmitters [16]. Therefore, it is crucial to maintain Phe levels within a safe range.

To engineer Phe sensors, we characterized the dimerization capabilities of the regulatory domain of human PAH [17] in the presence of Phe in different cell compartments and with different fusion partners. In the nucleus, this dimerization induced transgene expression in a dose-dependent manner, and the system was further optimized by error-prone mutagenesis to obtain a high-performance sensor module exhibiting strong activation at Phe concentrations higher than the physiological range (35–120 µM) [9, 15]. By connecting the engineered sensor to an effector module, we achieved a cell-autonomous closed-loop system, which we named PRO (Phe regulation orchestration), that is regulated by dynamic Phe levels. The effector module consisted of two Phe-degrading enzymes, interleukin 4 induced 1 (IL4I1) [18], naturally expressed in mammals, and bacterial Phe ammonia lyase (PAL), which degrades Phe into a nontoxic byproduct [19]. A monoclonal cell line stably expressing the circuit was confirmed to operate at elevated Phe levels, both in monolayer cultures and when encapsulated into alginate beads, while remaining inactive at physiological concentrations. This precise control ensures activation only in the presence of nonphysiological levels of Phe, avoiding risks associated with continuous enzyme expression, such as excessive depletion of this essential amino acid. Further testing in human whole-blood cultures confirmed that encapsulated PRO cells efficiently reduced Phe to physiological levels *ex vivo*. Finally, as proof of concept, we showed that alginate-encapsulated PRO cells implanted in a PKU mouse model significantly reduced blood Phe levels compared to those of control mice implanted with non-engineered cells.

## Materials and methods

### Plasmid construction

Supplementary Table S1 presents the design and cloning details for all genetic components utilized in this study. Plasmid design was performed using Benchling (www.benchling.com). Standard molecular cloning techniques were applied to obtain the desired plasmids. For restriction enzyme-based cloning, plasmids were digested with the appropriate endonucleases (New England Biolabs) and combined with the digested backbones, previously dephosphorylated with Quick CIP (M0525L, New England Biolabs), for ligation using T4 DNA ligase (EL0011, Thermo Fisher). For Gibson assembly, the sequences of interest and backbones were polymerase chain reaction (PCR)-amplified using primers that had ~20 bp complementary sequences at each end, digested with DpnI (R0176L, New England Biolabs) and cloned utilizing a custom mix of enzymes. To perform mutagenesis PCR, primers of variable length were designed to have the desired mutation/new sequence between a sequence complementary to the backbone and a sequence complementary to the other primer. Whole plasmid PCR amplification products were DpnI-digested before direct transformation into the *Escherichia coli* cloning strain. Primer sequences are reported in Supplementary Table S2. PCR reactions were performed using Q5 High-Fidelity DNA polymerase (M0491L, New England Biolabs). All plasmids were cloned and amplified in One shot TOP10 *E. coli* strain (C404010, Thermo Fisher) and DNA was extracted using a plasmid miniprep (ZR Plasmid Miniprep—Classic; D4054, Zymo Research) or midiprep (ZymoPURE II Plasmid Midiprep Kit; D4200, Zymo Research). Constructs were verified by Sanger sequencing performed by an external vendor (Microsynth AG). Synthetic gene fragments (Supplementary Table S3) used in the study were codon-optimized for expression in human cells and commercially synthesized by Twist Bioscience.

### Cell culture

Human embryonic kidney cells (HEK-293T, ATCC: CRL-3216) were cultivated in Dulbecco’s modified Eagle’s medium (DMEM, high glucose, GlutaMAX supplement; 61965026, Thermo Fischer) supplemented with 10% v/v fetal bovine serum (FBS; F7524, Sigma–Aldrich) under a humidified atmosphere containing 5% CO_2_ at 37°C. Passaging of preconfluent HEK-293 cultures was done by detaching cells through incubation in 0.05% trypsin–ethylenediaminetetraacetic acid (EDTA) (25300054, Life Technologies) for 3 min at room temperature. Cells were transferred to 10 ml culture medium, centrifuged for 2 min at 250 × *g*, resuspended in fresh medium, and reseeded at the desired cell density. Cell number and viability were quantified using a CellDrop automated cell counter (DeNovix). In the sensor and effector module experiments, cells were cultivated in a customized DMEM without l-phenylalanine, l-tryptophan, and l-tyrosine (Cell Culture Technologies, Switzerland), supplemented with 0.4 mM l-tyrosine, 0.078 mM l-tryptophan, and the indicated amount of l-phenylalanine for induction. During transfection, cells were supplemented with standard FBS-free DMEM.

### Transient transfection

At 24 hours before transfection in 96-well plates (3599, Corning), each well was seeded with 1–1.5 × 10^4^ cells. The transfection mixture consisted of 50 µl of FBS-free DMEM containing a 1:4 DNA:polyethylenimine (PEI, MW 40000; 24765, Polysciences) mixture, with a total DNA amount of 100–150 ng per well. After 20 min incubation, the transfection mixture was added dropwise to the cells, which were then incubated overnight. The culture medium was replaced with fresh DMEM supplemented with FBS, with or without the inducer of choice (specified in the figure legends). Supernatant samples were collected 24 hours later for analysis, unless otherwise indicated. Details of the transient transfections are presented in Supplementary Table S4.

### Exposure to inducers

Inducers were supplemented to cell culture media as indicated in the figures or figure legends. The following inducers were used: l-phenylalanine (P5482, Sigma–Aldrich), l-tyrosine (T8566, Sigma–Aldrich), l-tryptophan (T8941, Sigma–Aldrich), doxycycline hyclate (D9891, Sigma–Aldrich), RR120 (R0378, Sigma–Aldrich), xylose (X3877, Sigma–Aldrich), and rapamycin (553210, Sigma–Aldrich).

### Generation of monoclonal stable cell lines

Polyclonal cell lines were generated by cotransfecting HEK-293 cells with a hyperactive Sleeping Beauty transposase (pTS395) expression vector in a 1:10 (w/w) ratio with the indicated plasmids, containing SB recognition sites, puromycin or blasticidin resistance marker, and yellow fluorescent protein (YFP) or mRuby fluorophore. The medium was replaced 12 hours after transfection; cells were then incubated for 48 hours before the addition of DMEM supplemented with 2.5 µg ml^−1^ puromycin (ant-pr-1, Invivogen) and/or 5 µg ml^−1^ blasticidin (ant-bl-05, Invivogen). After 3–5 passages, the polyclonal population was sorted into single cells by FACS (fluorescence-activated cell sorting) in a 96-well plate format and expanded to obtain monoclonal cell lines.

### Secreted alkaline phosphatase quantification

Heat-inactivated (30 min, 65°C) cell culture supernatants (20 µl/sample) were mixed with 80 µl water, 80 µl 2 × SEAP (secreted alkaline phosphatase) buffer (20 mM homoarginine, 1 mM MgCl_2_, 21% v/v diethanolamine, pH 9.8), and 20 µl substrate solution with 20 mM para-nitrophenyl phosphate (pNPP; 128860100, Acros Organics BVBA). The absorbance of samples was measured at 405 nm using a Tecan Spark plate reader (Tecan AG) at 37°C.

### NanoLuc quantification

Secreted Nanoluc (NLuc) in cell culture supernatants was measured with the Nano-Glo Luciferase Assay System (N1110, Promega). Briefly, 7.5 µl from each sample was mixed with 7.5 µl buffer/substrate mix (50:1) in 384-well plates (781076, Greiner Bio One) and incubated at room temperature for 10 min. Luminescence was measured with a Tecan Spark plate reader. For analysis of intracellular NLuc, cells were lysed and NLuc measured following the manufacturer’s instructions.

### Phe quantification

Phe in cell culture medium, whole blood, and serum was measured with a Phe assay kit (EPHE-100, BioAssay Systems; MAK484, Sigma–Aldrich). Samples were deproteinized using 3–10 kDa ultracentrifugal filters. The assay was performed following the manufacturer’s instructions and measurements performed with a Tecan Spark plate reader.

### Western blot

Cell supernatants were used for extracellular protein expression. For intracellular expression, cells were harvested by centrifugation and washed twice with cold PBS. Cell lysis was performed with NP-40 buffer supplemented with protease inhibitor cocktail (10837091001, Sigma–Aldrich), and resuspended cells were left on ice for 30 min and vortexed occasionally, before centrifugation at 12 000 rpm for 20 min at 4°C. The cleared lysates and the cell culture supernatants were supplemented with 4 × Bolt™ LDS Sample Buffer (B0007, Invitrogen) and 1 mM dithiothreitol, incubated 5 min at room temperature, and boiled 10 min at 70°C. The samples were loaded on a 4%–12% Bis–Tris gel (XP04120BOX, Invitrogen) using 2-(N-morpholino)ethanesulfonic acid running buffer (MES; NP0002, Invitrogen). PageRuler™ Prestained Protein Ladder, 10–180 kDa (26616, Thermo Fischer) was used as a protein molecular weight ladder. Blotting was performed onto polyvinylidene difluoride (PVDF) membrane using a Biorad PowerPacTM. Blots were blocked with 10% skim milk in Tris-buffered saline with Tween 20 (TBST) buffer at room temperature for 30 min. The following antibodies were used: monoclonal anti-HA from mouse (H3663, Sigma–Aldrich) diluted 1:10 000 as the primary antibody incubated overnight at 4°C and HRP-conjugated anti-mouse antibody from sheep (NA931, Cytiva) diluted 1:10 000 as the secondary antibody. Blots were developed using Pierce™ ECL Western Blotting Substrate (32106, Thermo Fisher) and FUSION Pulse TS (37480003, Vilber, France). Images were analyzed using Adobe Photoshop software.

### Encapsulation of mammalian cells

(Engineered) HEK-293 cells were microencapsulated into coherent alginate-(poly-l-lysine)-alginate capsules (400 µM; 200 cells/capsule) using an Inotech Research IE-50R encapsulator (Buechi Labortechnik AG) with the following parameters: 20 ml syringe operated at a flow rate of 400 units, 200 µm nozzle with a vibration frequency of 1200 Hz, and bead dispersion voltage of 1200 V. The integrity and quality of the capsules were confirmed by microscopy.

### Whole-blood co-culture with encapsulated cells

Human whole blood from two healthy donors was collected at a blood donation center (Stiftung Blutspendenzentrum SRK beider Basel, Switzerland) in venous blood collection tubes containing heparin. The samples were used immediately for experimentation, following the protocol previously published by Schukur *et al.* [20]. Briefly, the encapsulated cells were resuspended in full DMEM supplemented with P/S (final 1%) and Phe (final concentration ~1 mM) to a total volume of 500 µl and added to 500 µl of human whole blood in 24-well plates. The cells were incubated under a humidified atmosphere containing 5% CO_2_ at 37°C. After 2 days of co-culture, fresh Phe-containing DMEM was supplemented. Thirty microliters of samples were taken every 24 hours for Phe quantification. The use of human blood samples for *ex vivo* blood culture is approved by the federal Ecogen database (notification number A172004-01).

### Animal experiments

B6.BTBR-*Pah^enu2^* mice were housed in a pathogen-free animal facility, in a temperature- and humidity-controlled room on a 12-h light–dark cycle with *ad-libitum* access to diet (9% protein; C1003, Altromin) and drinking water. The animals were bred in-house and genotyped at weaning. Homozygous animals (equal numbers of females and males in each group) were randomly assigned to experimental groups and i.p.-injected with ~5 × 10^6^ encapsulated cells. Blood samples were collected at the indicated days before (day 0) and post injection and after 4 hours fasting from the tail vein using BD Microtainer SST tubes according to the manufacturer’s instructions. In the control experiments, male C57BL/6JRI mice (Janvier LabsSaint-Berthevin, France) were used. All experiments involving animals were performed in accordance with Swiss animal welfare legislation, approved by the veterinary office of the Canton Basel-Stadt (approval no. 3200/35863) and carried out by Yu-Qing Xie with support from Marie-Didiêe Hussherr.

### Statistical analysis

Statistical evaluation was conducted by using an unpaired Student’s two-tailed *t*-test to compare two sets of data or one-way ordinary ANOVA for multiple comparisons as implemented in Prism GraphPad 9 (GraphPad Software Inc., San Diego, CA).

## Results

### Engineering and characterization of a Phe-responsive mammalian sensor

**Figure 1.**
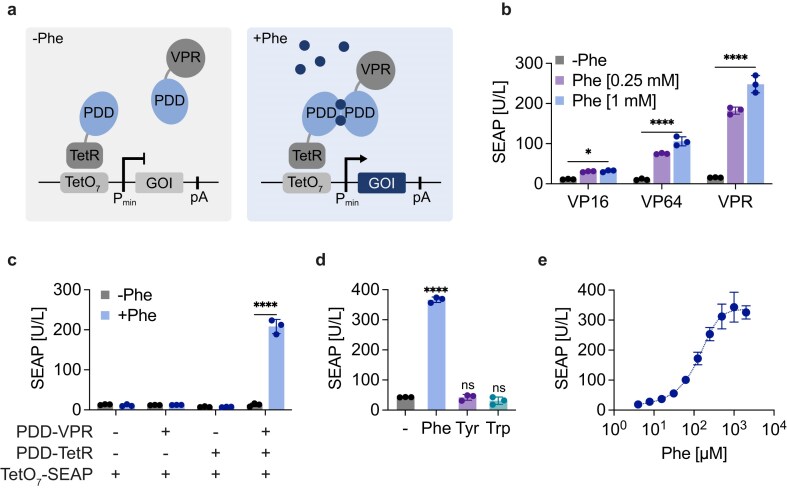
Engineering of a Phe-responsive gene switch in mammalian cells. (**A**) Schematic representation of the Phe-inducible gene switch in mammalian cells. In the presence of Phe, PDD–TetR and PDD–VPR colocalize in proximity to the synthetic TetR responsive promoter (TetO_7_-P_min_) and induce gene of interest expression. (**B**) Screening of different mammalian TAD. Cells transiently transfected with PDD–TetR, TetO_7_-P_min_–SEAP, and PDD C-terminally fused to VP16, VP64, or VPR, were cultured with 0, 0.25, or 1 mM Phe for 24 hours (**C**) Performance of the PDD gene switch. Cells transfected with TetO_7_-P_min_–SEAP along with constitutive expression of PDD–VPR or PDD–TetR, or both constructs were incubated with or without 1 mM Phe for 24 hours (**D**) Specificity of the gene switch for Phe over other natural aromatic amino acids. Cells transfected with the Phe gene switch were cultured for 24 hours with 1 mM Phe, 1 mM Tyr, or 1 mM Trp or without an excess of any of the aromatic amino acids. (**E**) Dose-response relationship of the gene switch. Cells transfected with the gene switch were incubated for 24 hours with the indicated Phe concentrations. The calculated EC50 value is ~133 µM. Data are shown as mean ± SD of *n* = 3 biologically independent samples (the individual data points are shown as dots). Statistical significance was calculated by two-tailed unpaired Student’s *t-*test (versus uninduced control) in panels (b) and (c), and ordinary one-way ANOVA (versus uninduced control) in panel (d); ns: not significant, *P < .05, ****P < .0001.

To engineer a mammalian sensor module regulated by Phe, we selected the regulatory domain of human PAH, located at the first 118 amino acids of the enzyme’s N-terminus [17, 21]. Structural and biochemical studies suggest that this domain (hereafter referred to as the Phe dimerization domain, PDD), which contains a structural motif characteristic of enzymes regulated by small-molecule ligands, undergoes homodimerization in the presence of Phe [22, 23]. We hypothesized that this property of PDD could be utilized to bring a DNA-binding domain (DBD) and a transactivation domain (TAD) into close proximity in the presence of Phe, thereby initiating gene expression from a promoter harboring cognate binding sites (Fig. 1A). Due to its strong affinity for TetO_7_ binding sites, we selected the tetracycline-responsive protein (TetR) as the DBD and we fused PDD to different mammalian TADs, i.e. VP16 (herpes simplex virus activation domain), VP64 (four tandem repeats of VP16), and VPR (VP64-p65-Rta tripartite fusion protein; VP64, p65, and the Epstein-Barr virus R transactivator). These constructs were transiently expressed in human embryonic kidney (HEK-293) cells, along with PDD fused to TetR and the reporter protein human placental SEAP controlled by a TetR-responsive synthetic promoter (TetO_7_-P_min_). Phe appears to induce dimerization of the PDD constructs, as expected, as all the tested TADs enabled increased reporter gene expression in a Phe-dependent manner (Fig. 1B). Cells constitutively expressing SEAP were grown with or without an excess of Phe as controls (Supplementary Fig. S1A). PDD fused to the strong transactivator VPR yielded the best fold-induction and highest expression of SEAP when cells were supplemented with Phe in the culture medium. A nuclear localization sequence (NLS) at the N-terminus of the PDD–VPR fusion protein was essential to ensure strong transgene expression (Supplementary Fig. S1B). Cells transfected with the reporter plasmid alone or with either one of the PDD constructs alone showed no SEAP expression in the presence of Phe (Fig. 1C). Indeed, the genetic switch is activated only when both dimerizing partners are expressed and an excess of Phe is present in the culture medium. We also tested the PDD from rat, which shares 85% homology at the amino acid level with human PDD. Although SEAP expression was significantly activated, greater responsiveness to Phe was observed with the human PDD (Supplementary Fig. S1C). Moreover, we tested a truncated version of PDD lacking the N-terminal 18 amino acid peptide^19–118^, as *in vitro* studies suggest that the terminal peptide is not essential for Phe binding [24]. While truncated PDD could promote transgene expression, its performance was inferior to that of full-length PDD (Supplementary Fig. S1D). Finally, we tested the specificity of the developed gene switch against two other aromatic amino acids, tyrosine (Tyr) and tryptophan (Trp). Neither Tyr nor Trp triggered significant reporter gene expression in engineered cells, which were confirmed to be activated by Phe, suggesting that the genetic switch is specific for Phe (Fig. 1D). The developed switch exhibited a concentration-dependent response to Phe, with an EC50 of ~133 µM (Fig. 1E), just above the normal physiological range.

### Characterization of Phe-inducible PDD dimerization in different cell compartments

**Figure 2.**
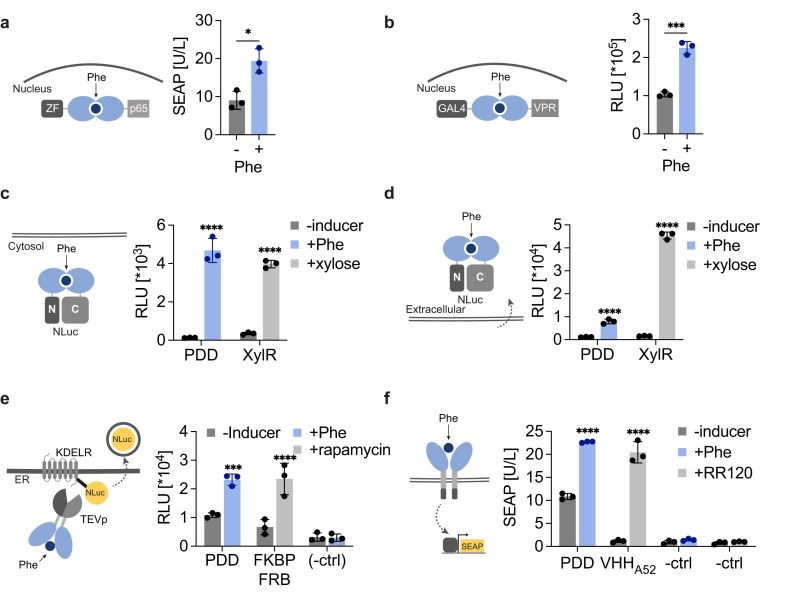
Characterization of PDD dimerization in different cellular contexts. Dimerization in the nucleus. (**A**) Cells were transfected with nuclear-localized PDD–ZF and PDD–p65, and with a reporter protein controlled by four binding sites specific for ZF binding. Engineered cells were cultured with or without 1 mM Phe for 24 hours (**B**) Cells were transfected with PDD–GAL4 and PDD–VPR, and with a reporter plasmid carrying NLuc controlled by GAL4 binding sites. (**C**) Dimerization in the cytosol. Cells transfected with PDD–NLuc^N^ and PDD–NLuc^C^, or with XylR–NLuc^N^ and XylR–CNluc^C^ as controls, were grown in the presence or absence of the inducers, 1 mM Phe and 5 mM xylose, respectively. After 24 hours intracellular NLuc was measured. (**D**) Extracellular dimerization. Cells were engineered to secrete PDD–NLuc^N^ and PDD–NLuc^C^, or XylR–NLuc^N^ and XylR–CNluc^C^ as controls, and cultured with or without the inducers, 1 mM Phe and 5 mM xylose, respectively. After 24 hours intracellular NLuc was measured. (**E**) Dimerization in the ER. Cells engineered with ER-localized PDD fusion to either part of a split TEV and with NLuc–TCS–KDEL were induced with or without 1 mM Phe for 30 min before NLuc analysis. As controls, cells were transfected with rapamycin dimerization domains fused to split TEV and induced for 30 min with 100 nM rapamycin. (-ctrl) indicates the transfection of NLuc–TCS–KDEL plasmid alone, with or without both inducers. (**F**) Dimerization on the plasma membrane. SEAP analysis of cells expressing the fusion transmembrane protein PDD–EpoR–IL6, STAT3, and SEAP controlled by STAT3-responsive promoter. VHH_A52_ antibody against RR120 (reactive red 120) was used as a control for membrane dimerization. Cells were cultured in the presence or absence of 1 mM Phe or 100 ng/ml RR120 for 24 hours (-ctrl) indicates cell transfected only with STAT3 and reporter plasmid. In all panels, the schematic of the assay is depicted on the left. Data are shown as mean ± SD of *n* = 3 biologically independent samples (the individual data points are shown as dots). Statistical significance was calculated by two-tailed unpaired Student’s *t-*test (versus uninduced control); *P < .05, ***P < .001, ****P < .0001.

To investigate the characteristics and potential applications of PDD, we examined its dimerization capabilities in other cellular contexts and with different fusion partners. First, we explored whether PDD-mediated dimerization could reconstitute the activity of other transcription factors when PDD was fused to different DBDs and TADs. PDD was N-terminally fused to an NLS for nuclear localization and C-terminally linked to either p65 or a synthetic zinc finger (ZF) designed to bind target DNA sequences [25]. These constructs were transiently transfected into HEK-293 cells in combination with a reporter plasmid coding for SEAP controlled by a ZF-responsive promoter (four ZF-specific DNA binding sites upstream of a minimal promoter). Significant SEAP expression was observed in the presence of Phe (Fig. 2A), although the performance was inferior to that of the Phe-inducible TetR-based gene switch or the constitutively expressed ZF-p65 control (Supplementary Fig. S1E). We also tested GAL4 as an additional orthogonal DBD partner. Increased reporter gene expression was observed when cells engineered with PDD–VPR and PDD–GAL4 were cultured in the presence of Phe (Fig. 2B). Next, to assess dimerization efficiency in the cytosol, PDD was fused to the N- and C-parts of a split NLuc luciferase [26]. In this scenario, we observed an increased NLuc signal in the presence of Phe, while no signal was detected in the absence of Phe (Fig. 2C). An analogous split-NLuc assay was implemented to test extracellular dimerization, with the key modification of adding a signal peptide for secretion at the N-terminus of the two fusion proteins. Significant NLuc activity was detected when Phe was added to the cell culture medium (Fig. 2D). For both assays, split-NLuc reconstitution mediated by XylR homodimerization in the presence of xylose was used as a control [27, 28]. Next, we tested PDD dimerization in the endoplasmic reticulum (ER) using a system that drives rapid secretion of preformed proteins retained in the ER [29]. Briefly, PDD fusions to two split fragments of an ER-residing tobacco etch virus protease (TEVp) variant were overexpressed along with NLuc tagged with KDEL for selective accumulation in the ER and a TEVp cleavage site (TCS) (KDEL–TCS–NLuc). Supplementation of Phe increased NLuc secretion in the supernatant, suggesting that PDD dimerized in the ER, thereby restoring TEVp activity and releasing the accumulated NLuc (Fig. 2E). The previously characterized rapamycin-inducible dimerization domains were included as a control. To test whether dimerization occurs on the extracellular side of the plasma membrane, we fused PDD to the C-terminus of EpoR in the GEMS receptor platform [30]. If extracellular dimerization occurs, the STAT3 (signal transducer and activator of transcription 3) endogenous pathway is activated, inducing reporter gene expression from a synthetic promoter containing STAT3-responsive elements. In the absence of the inducer, we observed some background SEAP expression, which increased when the engineered cells were treated with Phe (Fig. 2F). The inducible dimerization domain VHH_A52_ responsive to the small molecule RR120 was included as a control. Taken together, these results indicate that PDD dimerizes in the presence of Phe, irrespective of the cellular context. However, the efficiency of dimerization appears to be influenced by the nature of the cellular compartment and the fusion partners.

### Engineering of PDD for improved gene switch performance

**Figure 3.**
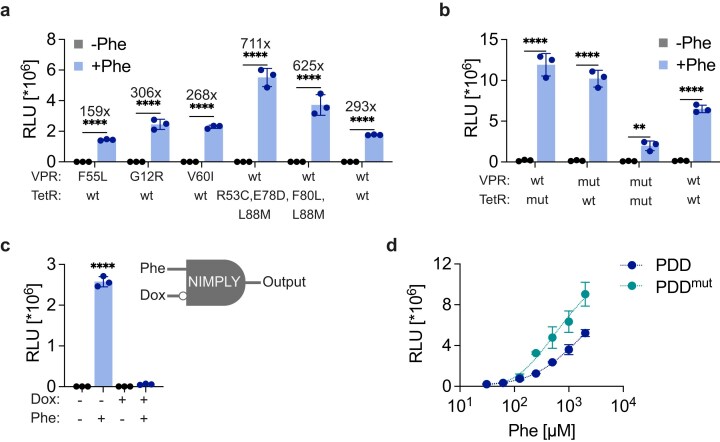
Optimization of the Phe genetic switch. (**A**) Comparison of the best mutant hits from both libraries. Cells were transfected with one wild-type PDD construct and one mutant PDD construct, as indicated in the figure. Numbers above the bars indicate fold difference in NLuc expression levels, calculated by dividing the mean reporter expression level in the presence of Phe by the mean expression level in the absence of Phe. (**B**) Effect of different combinations of PDD with PDD^m^, both fused to either VPR or TetR. Cells express either heterodimers or homodimers of PDD and PDD^m^ as fusion partners to VPR or TetR. (**C**) NIMPLY logic gate responsive to Phe and Dox. Cells expressing the Phe genetic switch were treated with 1 mM Phe and 1 µg/ml Dox as indicated in the figure. (**D**) Dose-dependent induction of NLuc with different concentrations of Phe. Cells transfected with both versions of the Phe genetic switch were incubated with the indicated Phe concentrations for 24 hours In panels (a–c), cells were incubated with or without 1 mM Phe for 24 hours before NLuc analysis. Data are shown as mean ± SD of *n* = 3 biologically independent samples (the individual data points are shown as dots). Statistical significance was calculated by two-tailed unpaired Student’s *t-*test (versus uninduced control) in panels (a) and (b), and ordinary one-way ANOVA in panel (c); **P < .01, ****P < .0001.

To establish an autonomous system responsive in the clinically relevant range for PKU patients, substantial activation of the genetic switch encoding a Phe-degrading protein should occur at Phe concentrations above 120 µM. Aiming for a more graded response with stronger induction, we performed error-prone PCR-based random mutagenesis on the PDD sequence. Two libraries, comprising 95 PDD–VPR mutants and 47 PDD-TetR mutants, were generated and screened in HEK-293 cells cotransfected with the wild-type PDD counterpart and a TetO_7_-inducible NLuc reporter construct. The cells were then incubated with or without Phe and assayed for NLuc secretion after 24 hours (Supplementary Fig. S2A and B). Primary and secondary screening yielded three PDD-VPR mutant hits that performed similarly to the wild type (Supplementary Fig. S2C) and seven PDD-TetR mutant clones whose performance was similar to or better than that of the wild type (Supplementary Fig. S2D). A side-by-side comparison of the top five mutants from both libraries showed that the best-performing PDD variant (PDD^m^) in terms of fold-induction and absolute reporter values has three mutations (R53C, E78D, L88M) spanning across the PDD sequence (Fig. 3A). These mutations seem to favor dimerization of PDD^m^, fused to either VPR or TetR, with its PDD counterpart (Fig. 3B). Therefore, we adopted PDD-VPR and PDD^m^-TetR as the heterodimerizing couple in all subsequent experiments. The new PDD^m^ was tested as a fusion to the three TADs with PDD-VPR, and it was confirmed that VPR is the best-performing TAD in the switch (Supplementary Fig. S3A). Furthermore, we observed that PDD^m^ performs similarly to or better than PDD in the nucleus, ER, and cytosol. This suggests that the substitution of glutamic acid with cysteine may influence subsequent post-translational modifications (Supplementary Fig. S3B–G). To further characterize the synthetic Phe sensor, we assessed whether it could be deactivated in the presence of doxycycline (Dox), which impairs the binding of TetR to its cognate promoter. Indeed, Dox overrides the Phe logic gate (A and not B), which is activated by A (Phe) if B (Dox) is not present, with the true state showing 109-fold higher response than the average of the false states (Fig. 3C). Notably, the Phe genetic switch is strongly induced in Phe concentration range clinically relevant to PKU with a wider dynamic range (Fig. 3D) and a more favorable EC50 (~278 µM) compared to the initial version of the genetic switch (Supplementary Fig. S3H). Thus, random mutagenesis afforded a superior PDD variant for an optimized Phe genetic switch, which is strongly activated by high Phe concentrations while remaining inactive in the absence of Phe or in the presence of low concentrations of Phe.

### Development of the effector module to deliver Phe-degrading enzymes

**Figure 4.**
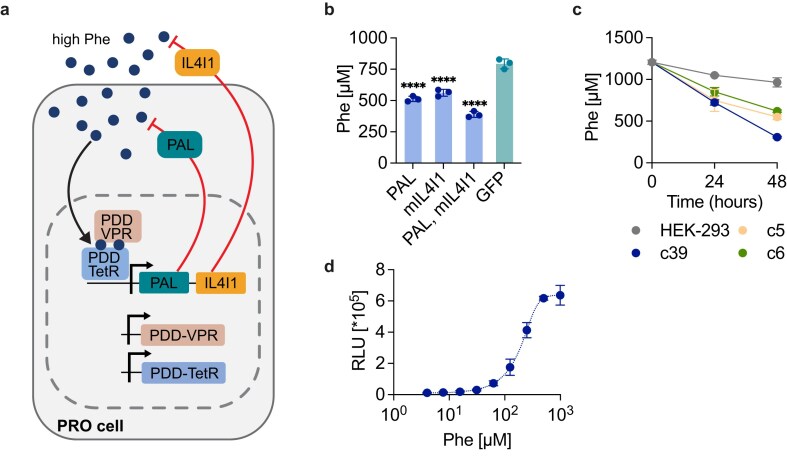
Generation of a Phe-dependent closed-loop system. (**A**) Schematic of the PRO closed-loop system. Elevated Phe levels activate the engineered sensor module (PDD-TetR and PDD-VPR) to trigger the expression of the effector module (Phe-degrading proteins PAL and IL4I1), creating a self-autonomous regulating system that balances Phe levels. (**B**) Phe consumption by Phe-degrading enzymes. Cells constitutively expressing either PAL, mIL4I1, both PAL and mIL4I1, or GFP were cultured with 1 mM Phe for 24 hours before analysis of Phe levels. (**C**) Time course of Phe consumption *in vitro*. Best-performing clones c39, c5, c6, and control HEK-293 cells, seeded at 0.6 × 10^6^ cells/ml in a 24-well plate, were cultured with high Phe for 48 hours. Phe concentration in the supernatants was measured at time points 0, 24, and 48 hours (**D**) Dose-response profile of PRO cells. PRO cells expressing TetO_7_-P_min_-NLuc responds dose-dependently to Phe. The calculated EC50 value is ~203 µM. Data are shown as mean ± SD of *n* = 3 biologically independent samples. Statistical significance was calculated by ordinary one-way ANOVA (versus GFP control) in panel (b); ****P < .0001.

Our next goal was to identify an efficient Phe-degrading effector protein to be expressed in response to the dose-dependent activation of the Phe genetic switch, thereby establishing a functional closed-loop system (Fig. 4A). We evaluated several candidate enzymes: (i) PAH, which converts Phe into Tyr; (ii) PAL, which degrades Phe into nontoxic *trans*-cinnamate [18]; and (iii) both human and mouse IL4I1 (hIL4I1 and mIL4I1), naturally secreted l-amino acid oxidases that catabolize Phe into phenylpyruvate, ammonia, and hydrogen peroxide [19]. Analysis of culture supernatants from cells constitutively expressing each enzyme revealed significantly reduced Phe levels in cells expressing PAL and mIL4I1 relative to control cells expressing a green fluorescent protein (GFP) (Supplementary Fig. S4A). Next, we tested PAL, mIL4I1, and a combination of both proteins at high Phe concentrations. PAL alone consumed ~35% of the supplemented Phe, while the combination of the two proteins increased Phe degradation to 51% (Fig. 4B). Moreover, no significant increase in H_2_O_2_, a possible toxic byproduct of IL4I1 activity, was detected in cell supernatants (Supplementary Fig. S4B). Western blot analysis confirmed the intracellular expression of PAL and the secretion of mIL4I1 (Supplementary Fig. S4C). Interestingly, mIL4I1 seems to be secreted to similar levels when using either its native secretion tag or the commonly used IgK signal peptide. Moreover, mIL4I1 truncations (mIL4I1^561^ and mIL4I1^509^) designed based on its predicted structure (PDB O09046) were less expressed in HEK-293 cell supernatants than the full-length protein, suggesting that the unstructured C-terminal portion is important for protein expression and stability. While constitutive expression of PAL and mIL4I1 tends to deplete Phe in the culture supernatant, Phe-inducible expression of these enzymes allows for controlled Phe metabolization up to a 200-µM threshold, effectively preventing complete depletion of this essential amino acid. This underscores the essential role of the sensor module in tightly regulating the enzyme expression (Supplementary Fig. S4D and E). Since PAL is a cytosolic enzyme, its intracellular expression alone in encapsulated therapeutic cells may not be sufficient to reduce blood Phe levels to the normal range. Therefore, we pursued two strategies for the therapeutic effector: mIL4I1 alone or in combination with PAL.

### Generation of stable PRO cells

To generate transgenic cell lines capable of autonomously controlling Phe levels, we combined the sensor and effector modules using different strategies: integrating both into a single plasmid or separating the sensor and effector modules into two plasmids. These plasmids were stably integrated in the cell genome using the Sleeping Beauty transposase system, generating three transgenic populations: (i) Phe-inducible mIL4I1 expressers, (ii) Phe-inducible dual mIL4I1and PAL expressers from a single plasmid, and (iii) dual expressers from two plasmids. After screening monoclonal cell lines isolated from each population (Supplementary Fig. S5A–C), we identified a clone expressing both effector proteins that outperformed the others, consuming ~90% of the Phe in the medium (Supplementary Fig. S5D). This clone (PRO cells) also showed superior Phe consumption over time (Fig. 4C) and was dose-dependently activated by Phe (Fig. 4D). Moreover, PRO cells displayed reversible transgene expression, cycling ON and OFF when alternated between Phe-containing and Phe-free media, indicating robust control over effector protein expression (Supplementary Fig. S5E). Therefore, PRO cells were selected for all subsequent experiments.

### Performance of encapsulated PRO cells *in vitro, ex vivo*, and *in vivo*

**Figure 5.**
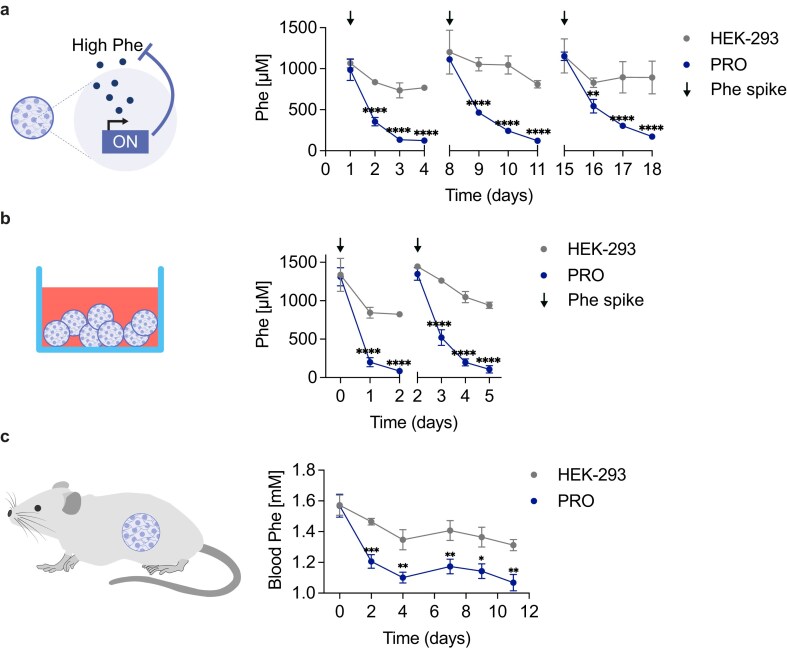
Performance of PRO cells *in vitro, ex vivo*, and *in vivo*. (**A**) *In vitro* Phe profile of encapsulated cells. PRO and HEK-293 cells were cultured and tested for Phe consumption over the course of three consecutive weeks. Every week, the capsules were washed and supplemented with fresh medium containing 1 mM Phe. Phe in the supernatant was measured every 24 hours for four consecutive days, each week. The experiment was started one week after encapsulation. (**B**) *Ex vivo* Phe profile of encapsulated cells co-cultured with human whole blood. Co-culture of PRO and HEK-293 cells in human whole blood from two different donors supplemented with 1 mM Phe at time points 0 and 2 days. Phe in the supernatant was measured every 24 hours for six consecutive days. (**C**) Phe profile of Pah^enu2^ mice implanted with encapsulated therapeutic or control cells. A baseline blood sample was taken at day 0 before i.p. injection (day 1) of microencapsulated therapeutic (PRO) or control (HEK-293) cells. Subsequently, blood samples were taken at days 2, 4, 7, 9, and 11 post-injection for Phe analysis. In panel (a), data are shown as mean ± SD of *n* = 3 biologically independent samples. In panel (b), data are shown as mean ± SD of *n* = 6 biologically independent samples. In panel (c), data are shown as mean ± SEM of *n* = 6 (3 males and 3 female) mice for the HEK-293 control and *n* = 8 (4 males and 4 females) mice for PRO. Statistical significance was calculated by two-tailed unpaired Student’s *t-*test; *P < .05, **P < .01, ***P < .001, ****P < .0001.

Alginate encapsulation is widely employed for implanting therapeutic cells in both preclinical and clinical studies, as it protects cells from the host immune response while allowing the diffusion of essential nutrients to support cell survival [31, 32]. Alginate-encapsulated PRO cells (Supplementary Fig. S6A) cultured *in vitro* exhibited a significant decrease in Phe levels within four days and this was sustained over the course of three weeks (Fig. 5A). In contrast, Phe levels remained elevated in cultures with encapsulated non-engineered HEK-293 cells. We subsequently assessed whether PRO cells could effectively function in a more physiologically relevant setting by co-culturing the encapsulated cells with fresh human whole blood. This *ex vivo* model constitutes a valuable alternative for assessing therapeutic cell performance without the complexity of animal experiments [20]. Encapsulated PRO cells were co-cultured in whole blood from two separate donors, with excess Phe added on days 0 and 2 (Fig. 5B). The experimental duration was limited to 5 days, as whole-blood cultures typically degrade within a few days [20, 33–35]. The Phe levels were measured every 24 hours, and the results showed significant Phe consumption by PRO cells compared to control cells. Although there were slight differences in baseline Phe levels between the donors (Supplementary Fig. S6B), the consumption profile remained consistent across both donors (Supplementary Fig. S6C and D), underscoring the robustness of the system in a human-like environment. Encouraged by these promising *in vitro* and *ex vivo* results, we conducted a proof-of-concept study in Pah^enu2^ mice, validated as a PKU mouse model [36]. These mice are homozygous for a single point mutation in the *Pah* gene, exhibiting blood Phe levels that are over 20-fold higher than those of heterozygous or wild-type controls (Supplementary Fig. S6E). Moreover, wild-type mice with or without the cell implant showed no significant difference in Phe levels, confirming that low Phe concentrations do not trigger PRO cells (Supplementary Fig. S6F). The mice were intraperitoneally (i.p.) injected with encapsulated therapeutic or control cells, after collection of a baseline blood sample. By day 2 post-implantation, mice injected with PRO cells showed a ~25% reduction in blood Phe compared to the baseline levels and a significantly lower blood Phe level than in control mice was maintained in the following days (Fig. 5C). Blood Phe levels stabilized at around 1.1 mM in treated mice. The control group also showed a slight decrease in Phe, likely due to baseline consumption of Phe by the encapsulated HEK-293 cells (Supplementary Fig. S6G). The observed ~400 µM decrease in Phe, if extrapolated to humans, would be sufficient to meet the human therapeutic target of a Phe concentration below 600 µM for many mild PKU cases (Phe levels 600–1200 µM) [14, 15, 37]. However, for severe PKU (above 1200 µM), further optimization of effector protein expression will likely be required. Nonetheless, this proof-of-concept experiment lays a strong foundation for system optimization and may inspire future therapeutic applications.

## Discussion

A central goal of synthetic biology is the development of advanced tools for next-generation cell-based therapies, moving toward safer, more personalized treatments for chronic diseases [38]. Cell-autonomous closed-loop systems represent a particular promising approach, as they can self-regulate, bypassing the need for external intervention. In this work, we have developed the first synthetic mammalian closed-loop system designed to sense and degrade elevated levels of Phe, laying the foundation for a new therapeutic strategy targeting PKU. Current PKU treatments are limited and largely ineffective at achieving metabolic control. Available therapies include: (i) the oral intake of sapropterin, an analog of the PAH cofactor tetrahydrobiopterin (BH_4_), which stabilizes only some PAH mutants and is used in combination with dietary management [39], and (ii) the systemic injection of pegvaliase, a bacterial PAL, which carries the risk of inducing severe immune reactions [15, 40, 41]. Ongoing research has been focused on developing gene therapy approaches to replace or correct the defective PAH gene [42–48]. However, addressing PKU through gene therapy remains a significant challenge due to both the current limitations associated with the technology [49] and the broad spectrum of mutations in the PAH gene [9, 50]. Furthermore, a promising strategy involving an *E. coli* Nissle strain engineered to promote Phe degradation [51] failed to show efficacy in phase 3 trials. Thus, therapeutic options for PKU are still limited or at an early stage of development. Indeed, strict dietary control remains the primary approach, but patient compliance in everyday life is often poor [39, 52–54].

Our approach relies on a Phe-inducible sensor module composed of the regulatory domain of human PAH, fused either to a strong transactivator or to a known DNA binding domain. In response to Phe, the regulatory domains dimerize, colocalizing the two fusion proteins at a synthetic promoter, thereby promoting effector gene expression. The system was shown to be dose-dependently activated by Phe but not by other aromatic amino acids. Moreover, PDD dimerization capability was confirmed in different cell compartments, namely the nucleus, ER, cytosol, plasma membrane, and extracellular space, with different fusion partners, showcasing the flexibility of this domain for future applications. By randomly mutating PDD in conjunction with phenotypic screening, we found a triple mutant PDD that, as a heterodimer with wild-type PDD counterpart, outperformed the original homodimerizing pair, exhibiting strong activation by Phe in the clinically relevant concentration range. The effector module consists of two Phe-degrading enzymes (IL4I1 and PAL), which degrade Phe into nontoxic byproducts. We generated a stable cell line with genomic integration of both sensor and effector modules, enabling dynamic Phe reduction *in vitro*. Therapeutic cells microencapsulated in alginate beads lowered Phe to healthy levels both *in vitro* (~120–180 µM) and *ex vivo* (~80–110 µM) in co-cultures with human whole blood.

In the *in vivo* proof-of-concept study, Pah^enu2^ mice—a stringent PKU model with Phe levels exceeding 20-fold the normal range—were given i.p. implants of encapsulated PRO cells. Treated mice displayed significantly lower Phe levels than the controls. In humans, mild PKU typically results in Phe levels between 600–1200 µM (5–10-fold increase) while severe cases exceed 1200 µM (10–15-fold increase) [9, 55]. The observed ~400 µM reduction in blood Phe in treated mice, if translatable to humans, would be sufficient to lower the Phe levels in many patients with mild PKU to below the 600 µM threshold that is considered safe [14]. Although standard practice in validating proof-of-concept closed-loop therapies for the treatment of experimental medical conditions [3, 4, 56, 57], the duration of the present *in vivo* animal experiment was still relatively short, for ethical and regulatory reasons. To further investigate the feasibility of clinical translation, it will be necessary to confirm longer-term noninferiority of the present closed-loop system to an existing treatment regimen, such as repeated systemic injection of pegvaliase [41, 58]. Alginate encapsulation is well established to protect engineered cells from host immune responses, but a more detailed characterization of the longevity of the engineered cells and the safety of the system will also be required [31, 59].

A crucial feature of our system is the sensor module, designed to activate effector expression only in response to pathological levels of Phe. This self-regulating response is important to (i) prevent the depletion of this essential amino acid, which can lead to severe complications [16], (ii) dynamically regulate the expression of therapeutic proteins, and (iii) simulate the natural PAH activity, which is tightly controlled to avoid constitutive enzyme activation [24]. While PRO cells demonstrated promising Phe-degrading capacity, further optimization is likely necessary to achieve target levels below 600 µM for severe PKU cases [37, 60]. Potential optimization strategies include expressing the PDD fusion proteins from weaker promoters to reduce competition for cellular resources and allow higher enzyme expression, and engineering Phe-degrading enzymes for enhanced performance.

In conclusion, this study presents a synthetic biology-based platform that integrates sensor–effector modules to achieve dynamic Phe regulation. Our findings suggest the potential of this approach as a next-generation cell-based therapy for PKU, which has thus far been only partially managed by conventional drugs. Moreover, we believe this sensor–effector framework could be adapted to address other metabolic disorders characterized by toxic metabolite accumulation, such as branched amino acids in maple syrup urine disease, Tyr in hypertyrosinemia, or ammonia in hyperammonemia.

## Supplementary Material

gkaf1151_Supplemental_File

## Data Availability

All plasmids reported in Supplementary Table S1 and data generated in this study are available on request. Requests for materials should be made to the corresponding author.
