# Supplementary material for: Synthetic closed-loop gene circuit for phenylalanine regulation
Source: Nucleic Acids Res. 2025 Nov 4;53(20):gkaf1151. doi: 10.1093/nar/gkaf1151 (PMC12585910; doi:10.1093/nar/gkaf1151)
Supplement: gkaf1151_Supplemental_File [file gkaf1151_supplemental_file.docx]

Supporting Information

**Synthetic closed-loop gene circuit for phenylalanine regulation**

Silvia Galvan^1^, Yuqing Xie^1^, Ana P. Teixeira^1^, Martin Fussenegger^1,2*^

^1^Department of Biosystems Science and Engineering, ETH Zurich, Klingelbergstrasse 48, CH-4056 Basel, Switzerland.

^2^Faculty of Science, University of Basel, Klingelbergstrasse 48, CH-4056 Basel, Switzerland.

^*^Corresponding author. E-mail: fussenegger@bsse.ethz.ch

**Contents**

1. **Supporting Figures S1-S6**
2. **Supporting Tables S1-S4**

**Supporting figures**

**Figure S1**

**Figure S1. a)** Effect of excess Phe on cells constitutively expressing SEAP. **b)** Effect of NLS on PDD-VPR fusion protein. Cells were co-transfected with TetO_7_-P_min_-SEAP, PDD-TetR and either PDD-VPR (PDD) or NLS-PDD-VPR (NLS). (-ctrl) indicates the transfection of TetO_7_-P_min_-SEAP and an empty plasmid (Filler) to make the same total DNA concentration. **c)** Comparison of the performance of human PDD (hPDD) and rat PDD (rPDD) Phe genetic switches. Cells were co-transfected with TetO_7_-P_min_-SEAP and either the hPDD or rPDD fusion proteins. **d)** Effect of the N-terminal 18 aa peptide on the performance of PDD in the Phe genetic switch. Cells were transfected with TetO_7_-P_min_-SEAP and either PDD-VPR/PDD-TetR or PDD^19-118^-VPR/PDD^19-118^-TetR. **e)** Constitutive expression of ZF-p65 fusion protein. Cells were transfected with the reporter plasmid and either NLS-ZF-p65, PDD-ZF or an empty vector (-ctrl). In all experiments, cells were incubated for 24 hours with or without 1 mM Phe (otherwise indicated) before SEAP analysis. Data are shown as mean ± SD of n = 6 (**a**) or n = 3 (**b**, **c**, **d**, **e**) biologically independent samples (the individual data points are shown as dots). Statistical signiﬁcance was calculated by two-tailed unpaired Student’s *t-*test. ns: not signiﬁcant, **P < 0.01, ****P < 0.0001.

**Figure S2**

**Figure S2.** Primary screening of PDD-VPR (**a**) and PDD-TetR (**b**) mutants generated by error-prone PCR. **a**) Cells were transfected with TetO_7_-P_min_-NLuc, PDD-TetR, and a mutant PDD-VPR. **b**) Cells were transfected with TetO_7_-P_min_-NLuc, PDD-VPR, and a mutant PDD-TetR. The dark blue dot is the wild-type control for both constructs. **c)** Secondary screening of the best-performing mutant variants from the PDD-VPR random mutagenesis library. **d)** Secondary screening of the best-performing mutant variants from the PDD-TetR random mutagenesis library. In all panels, cells were incubated with or without 1 mM Phe for 24 hours before NLuc analysis, unless indicated otherwise. In panels **c**, **d** data are shown as mean ± SD of n = 3 biologically independent samples (the individual data points are shown as dots). Statistical signiﬁcance was calculated by two-tailed unpaired Student’s *t-*test. **P < 0.01, ****P < 0.0001.

**Figure S3**

**Figure S3. Characterization of PDD^m^ dimerization in different cellular contexts. a)** Screening of different mammalian transactivation domains. Cells were transiently transfected with PDD^m^-TetR, TetO_7_-P_min_-SEAP and PDD C-terminally fused to either VP16, VP64, or VPR. **b)** Dimerization in the nucleus. Cells were transfected with homodimers or heterodimers of PDD and PDD^m^ fused to either ZF or p65 **c)** Dimerization in the nucleus. Cells were transfected with PDD^m^-GAL4 and PDD-VPR, and with a reporter plasmid carrying NLuc controlled by GAL4 binding sites. (-ctrl) indicates the reporter plasmid alone. **d)** Dimerization in the cytosol. Transfected cells with homodimers or heterodimers of PDD and PDD^m^ fused to either NLuc^N^ and NLuc^C^. **e)** Extracellular dimerization. Cells were engineered to secrete PDD and PDD^m^ fused to either NLuc^N^ and NLuc^C^ as homodimers or heterodimers. **f)** Dimerization in the ER. Cells engineered with NLuc-TCS-KDEL and an ER-localized PDD or PDD^m^ fusion to either part of a split TEV were induced for 30 min before NLuc analysis. **g)** Dimerization on the plasma membrane. SEAP analysis of cells expressing the fusion transmembrane protein PDD^m^-EpoR-IL6 alone or in combination with PDD-EpoR-IL6, STAT3 and SEAP controlled by a STAT3-responsive promoter. **h)** Dose-dependent induction of NLuc by Phe. Cells transfected with the optimized Phe genetic switch were incubated with the indicated Phe concentrations for 24 hours. The calculated EC50 value is ~278 µM. In all panels, cells were supplemented with or without 1 mM Phe for 24 hours, unless otherwise indicated. Data are shown as mean ± SD of n = 3 biologically independent samples (the individual data points are shown as dots). ﻿ Statistical signiﬁcance was calculated by two-tailed unpaired Student’s *t-*test; ns: not signiﬁcant, *P < 0.05, **P < 0.01, ***P < 0.001, ****P < 0.0001.

**Figure S4**

**Figure S4. a)** Phe concentration in supernatants of cells constitutively expressing Phe-degrading enzymes. GFP-transfected cells (green bar) are the controls. **b)** H_2_O_2_ levels in supernatants of cells constitutively expressing Phe-degrading enzymes. **c)** ﻿Immunoblotting of the HA tag of cells transfected with: PAL (lane 1: intracellular, expected MW~59 kDa), mIL4I1 (lane 2: supernatant, expected MW~72 kDa), ss-mIL4I1 (lane 3: supernatant, expected MW~72 kDa), mIL4I1^561^ (lane 4: supernatant, expected MW~64 kDa), mIL4I1^509^ (lane 5: supernatant, expected MW~58 kDa) or an empty plasmid (lane 6). Effect of Phe-regulated expression of PAL (**d**) and mIL4I1 (**e**) enzymes. Cells were engineered to express the Phe-degrading enzymes either constitutively (gray) or regulated by the Phe sensor (blue); GFP-transfected cells (green) are the controls. Remaining Phe levels were measured every 12 hours. In all panels, cells were cultured in full DMEM for 24 hours, otherwise indicated, before analysis. Data are shown as mean ± SD of n = 4 (**a**, **b**) or n = 3 (**d**, **e**) biologically independent samples (the individual data points are shown as dots). Statistical signiﬁcance was calculated by ordinary one-way ANOVA (vs GFP control). ns: not signiﬁcant, **P < 0.01, ****P < 0.0001.

**Figure S5**

**Figure S5. Generation of monoclonal transgenic cell lines.** Screening of sorted monoclonal cell lines of: Phe-inducible mIL4I1 expressers (pSG344) **(a)**; Phe-inducible dual mIL4I1 and PAL expressers from a single plasmid (pSG346) **(b)** or double plasmids (pSG372 and pSG508) **(c)**. Cells were grown with or without 1 mM Phe, the green dots represent the fold change between induced and non-induced conditions. **d)** Phe consumption ability of the best hits selected from **a**, **b**, **c**. Cells were incubated with 1 mM Phe for 48 hours before Phe analysis. Non-transgenic HEK-293 cells were used as controls**. e)** Reversibility of PRO cell activation. Cells were cultivated for 96 hours and the medium was exchanged every 24 hours, alternating between medium containing 400 µM Phe and no Phe. Data are shown as mean ± SD of n = 3 biologically independent samples (the individual data points are shown as dots). Statistical signiﬁcance was calculated by ordinary one-way ANOVA (vs HEK-293 control). ****P < 0.0001.

**Figure S6**

**Figure S6. Encapsulated therapeutic (PRO) and control (HEK-293) cells.** **a)** Microscopy imaging (10X) of PRO cells. Left panel shows the bright-field image, right panel shows the expression of fluorescent YFP. **b)** Comparison of the levels of Phe between donors A and B at time point 0. The two whole blood samples were supplemented with 1 mM Phe to co-culture the encapsulated cells. **c)** Phe consumption profile of donor A. **d)** Phe consumption profile of donor B. **e)** Phe levels of wild type and Pah^enu2^ mice with the indicated genotype. **f)** Effect of PRO cell implant on baseline levels of Phe. Phe levels of wild type mice with or without i.p. implant of encapsulated PRO cells 2 days post-injection. **g)** Phe levels in PKU mice implanted with encapsulated HEK-293 cells. In panels **c**, **d** and **e** data are shown as mean ± SD of n = 3 biologically independent samples. In panel **f** data are shown as mean ± SEM of n = 4 biologically independent samples. Statistical signiﬁcance was calculated by two-tailed unpaired Student’s *t-*test. In panel **g** data are shown as mean ± SEM of n = 6 biologically independent samples. Statistical signiﬁcance was calculated by one-way ANOVA for repeated measurements. ns: not signiﬁcant, *P < 0.05.

**Supporting Tables**

**Table S1 | Plasmids used in this work**

| **ID** | **Detailed Cloning Description** | **Source** |
| --- | --- | --- |
| pAna225 | Vector coding for GAL4DBD-dependent NLuc expression with a sTRSV ribozyme-dependent destabilization module.  (P5xUAS-ss-NLuc-sTRSV-p_AbGH_) | Franko et al., 2021 |
| pFox12 | Vector coding for constitutive expression of eGFP.  (P_hCMV_-eGFP-pA_bGH_) | Haellman et al., 2021 |
| pGU137 | Vector coding for p2A-SEAP  (P-coding region-p2A-SEAP-p_AbGH_) | Unal et al, unpublished |
| pLeo619 | Vector coding for constitutive expression of VHH_A52_-EpoR_m_-IL6RB_m_  (P_SV40_-ss-VHH_A52_-EpoR_m_-IL6RB_m_-p_AbGH_) | Scheller et al, 2018 |
| pLS13 | Vector coding for expression of SEAP driven by a STAT3-responsive promoter.  (P_STAT3_-SEAP-pA_bGH_) | Schukur et al. 2015 |
| pLS15 | Vector coding for constitutive expression of STAT3.  (P_hCMV_-STAT3-pA_bGH_) | Schukur et al. 2015 |
| pMMH26 | Vector coding for constitutive expression of an ER-localized split TEV protease fused to FKBP dimerization domain. (P_hCMV_-ss-FKBP-(GGGGS)_2_-ssTEVp_1-118_-KDEL-pA_bGH_) | Mahameed et al. 2022 |
| pMMH27 | Vector coding for constitutive expression of an ER-localized split TEV protease fused to FRB dimerization.  (P_hCMV_-ss-FRB-(GGGGS)_2_-ssTEVp_119-245_-KDEL-pA_bGH_) | Mahameed et al. 2022 |
| pMMH91 | Vector coding for constitutive expression of NLuc-KDEL with TCS.  (P_hCMV_-NLuc-TCS-KDEL-pA_bGH_) | Mahameed et al. 2022 |
| pOM219 | Vector coding for constitutive expression of XylR-VPR  (P_hCMV_-NLS-XylR-VPR-pA_bGH_) | Galvan et al. 2022 |
| pTS395 | Vector coding for constitutive expression of Sleeping Beauty transposase.  (P_hCMV_-SB100-pA_bGH_) | Haellman et al., 2021 |
| pTS1012 | Vector encoding constitutive expression of secreted NLuc reporter protein.  (P_hCMV_-ss-NLuc- pA_bGH_) | Scheller et al, 2018 |
| pTS1017 | Vector encoding O_TetO7_-driven SEAP reporter protein.  (O_TetO7_-P_min_-SEAP-pA_bGH_) | Haellman et al., 2021 |
| pTS1022 | Vector encoding P_hCMV_-driven SEAP reporter protein.  (P_hCMV_-SEAP-pA_bGH_) | Haellman et al., 2021 |
| pTS1029 | Tier-3 vector for stable integration of up to two cassettes via Sleeping Beauty transposase, encodes YPet-p2A-PuroR for selection.  (5’ITR-cassette1-pA_bGH_::cassette2-pA_p36_::P_RPBSA_-YPet-p2A-PuroR-pA_p9_-3’ITR) | Haellman et al., 2021 |
| pTS1107 | Tier-3 vector for stable integration of up to three cassettes via Sleeping Beauty transposase.  (5’ITR-cassette1-pA_bGH_::cassette2-pA_p36_::cassette3-pA_p9_-3’ITR) | Haellman et al., 2021 |
| pTS1214 | P_hCMV_-driven expression vector with self-cleaving p2A peptide between MCSs.  (P_hCMV_-MCS-p2A-MCS-pA_bGH_) | Strittmatter et al.  unpublished |
| pTS2344 | Tier-3 vector for stable integration of up to two cassettes via Sleeping Beauty transposase, encodes mRuby2-p2A-BlastR for selection.  (5’ITR-cassette1-pA_bGH_::cassette2-pA_p36_::P_RPBSA_-BlastR-p2A-mRuby2-pA_p9_-3’ITR) | Haellman et al., unpublished |
| pTS2365 | Vector coding for constitutive expression of TetR.  (P_hCMV_-TetR-pA_bGH_) | Haellman et al., unpublished |
| pTS2367 | Vector encoding constitutive expression of VP16.  (P_hCMV_-VP16-pA_bGH_) | Strittmatter et al.  unpublished |
| pTS2368 | Vector encoding constitutive expression of VP64.  (P_hCMV_-VP64-pA_bGH_) | Strittmatter et al.  unpublished |
| pTS2373 | Vector encoding constitutive expression of GAL4DBD.  (P_hCMV_-GAL4DBD-pA_bGH_) | Haellman et al., unpublished |
| pVH15 | Vector encoding constitutive expression of VPR.  (P_hCMV_-VPR-pA_bGH_) | Haellman et al., unpublished |
| HSCD00288181 | <https://dnasu.org/DNASU/GetCloneDetail.do?cloneid=288181#sequence>  Vector coding for hPAH produced by the ORFeome Collaboration. | Seiler et al., 2014 |
| pSG015 | Vector coding for a constitutively secreted hPAH.  (P_hCMV_-ss-hPAH-pA_bGH_)  hPAH was PCR-amplified from HSCD00288181 with the oligos oSG022-oSG023, digested with BsmBI, and ligated into pTS1012 PCR-amplified with oSG012-oSG013 and digested with BsmBI. | This work |
| pSG096 | Vector coding for constitutive expression of PDD-VPR.  (P_hCMV_-PDD-VPR-pA_bGH_)  The construct was cloned by Gibson assembly. The PDD sequence was PCR-amplified from pSG015 with oligos oSG098-oSG099; the backbone was PCR-amplified with oligos oSG007-oSG106 from pVH15. | This work |
| pSG097 | Vector coding for a constitutive expression of PDD^19-118^-VPR.  (P_hCMV_-PDD^19-118^-VPR-pA_bGH_)  The construct was cloned by Gibson assembly. The PDD^19-118^ sequence was PCR-amplified from pSG015 with oligos oSG099-oSG101; the backbone was PCR-amplified with oligos oSG007-oSG106 from pVH15. | This work |
| pSG100 | Vector encoding O_TetO7_-driven NLuc reporter protein.  (O_TetO7_-P_min_-NLuc-pA_bGH_)  NLuc was restricted from pTS1012 with EcoRI/HindIII and ligated into pTS1017 restricted with EcoRI/HindIII. | This work |
| pSG138 | Vector coding for constitutive expression of nuclear-localized PDD-VPR.  (P_hCMV_-NLS-PDD-VPR-pA_bGH_)  The NLS sequence was added by PCR amplification of pSG096 with oligos oSG032-oSG166. | This work |
| pSG140 | Vector coding for constitutive expression of nuclear-localized PDD^19-118^-VPR.  (P_hCMV_-NLS-PDD^19-118^-VPR-pA_bGH_)  The NLS sequence was added by PCR amplification of pSG097 with oligos oSG032-oSG167. | This work |
| pSG153 | Vector coding for nuclear localized PDD-GAL4DBD.  (P_hCMV_-NLS-PDD-GAL4DBD-pA_bGH_)  GAL4DBD was isolated from pTS2373 with SpeI/HindIII and ligated into pSG138 restricted with SpeI/HindIII. | This work |
| pSG199 | Vector coding for a constitutive PDD^19-118^-TetR expression cassette.  (P_hCMV_-PDD^19-118^-TetR-pA_bGH_)  pSG240 was PCR-amplified with oligos oSG007-oSG101. | This work |
| pSG214 | Vector coding for constitutive expression of XylR-NLuc^N^.  (P_hCMV_-XylR-NLuc^N^)  XylR was PCR-amplified from pOM219 with oligos oSG029-oSG246 and restricted with EcoRI/SpeI. NLuc^N^ was PCR-amplified with oligos oSG238-oSG248 from a gene fragment carrying NLuc^N^ and restricted with SpeI/HindIII. The restricted fragments were ligated into pTS1022 restricted with EcoRI/HindIII. | This work |
| pSG215 | Vector coding for constitutive expression of XylR-NLuc^C^.  (P_hCMV_-XylR-NLuc^C^)  XylR was PCR-amplified from pOM219 with oligos oSG029-oSG246 and restricted with EcoRI/SpeI. NLuc^C^ was obtained by oligos dimerization of oSG239-oSG240. The restricted fragments were ligated into pTS1022 restricted with EcoRI/HindIII. | This work |
| pSG227 | Vector coding for constitutive hPAH expression.  (P_hCMV_-hPAH-pA_bGH_)  pSG015 was PCR-amplified with oligos oSG007-oSG179. | This work |
| pSG230 | Vector coding for cytosolically expressed PDD-NLuc^N^.  (P_hCMV_-PDD-NLuc^N^-pA_bGH_)  The construct was cloned by Gibson assembly. NLuc^N^ was PCR-amplified from a commercially synthesized gene fragment with oligos oSG238-oSG248; the backbone was PCR-amplified with oligos oSG247-oSG250 from pSG240. | This work |
| pSG231 | Vector coding for a cytosolically expressed PDD-NLuc^C^. (P_hCMV_-PDD-NLuc^C^-pA_bGH_)  NLuc^C^ was obtained by oligos dimerization of oSG239-oSG240, ligated into pSG240 PCR-amplified with oligos oSG063-oSG251 and restricted with NheI/HindIII. | This work |
| pSG233 | Vector coding for constitutive HA-hPAH expression.  (P_hCMV_-HA-hPAH-pA_bGH_)  pSG227 was PCR-amplified with oligos oSG254-oSG256. | This work |
| pSG234 | Vector coding for constitutive PDD^F55L^-VPR.  (P_hCMV_-NLS-PDD^F55L^-VPR-pA_bGH_)  The mutant PDD was obtained by error-prone mutagenesis PCR on pSG138 using oligos oSG029-oSG253, digested with EcoRI/SpeI and inserted into pSG138 restricted with EcoRI/SpeI. | This work |
| pSG235 | Vector coding for constitutive PDD^G12R^-VPR.  (P_hCMV_-NLS-PDD^G12R^-VPR-pA_bGH_)  The mutant PDD was obtained by error-prone mutagenesis PCR on pSG138 using oligos oSG029-oSG253, digested with EcoRI/SpeI and inserted into pSG138 restricted with EcoRI/SpeI. | This work |
| pSG236 | Vector coding for constitutive PDD^V60I^-VPR.  (P_hCMV_-NLS-PDD^V60I^-VPR-pA_bGH_)  The mutant PDD was obtained by error-prone mutagenesis PCR on pSG138 using oligos oSG029-oSG253, digested with EcoRI/SpeI and inserted into pSG138 restricted with EcoRI/SpeI. | This work |
| pSG237 | Vector coding for constitutive PDD^F55L^-TetR.  (P_hCMV_-PDD^F55L^-TetR-pA_bGH_)  PDD^F62L^ expression cassette was isolated from pSG234 by EcoRI/HindIII restriction and ligated into pSG240 restricted with EcoRI/HindIII. | This work |
| pSG238 | Vector coding for constitutive PDD^G12R^-TetR.  (P_hCMV_-PDD^G12R^-TetR-pA_bGH_)    PDD^G12R^ expression cassette was isolated from pSG234 by EcoRI/HindIII restriction and ligated into pSG240 restricted with EcoRI/HindIII. | This work |
| pSG239 | Vector coding for constitutive PDD^V70I^-TetR.  (P_hCMV_-PDD^V70I^-TetR-pA_bGH_)  PDD^V70I^ expression cassette was isolated from pSG234 by EcoRI/HindIII restriction and ligated into pSG240 restricted with EcoRI/HindIII. | This work |
| pSG240 | Vector coding for a constitutive PDD-TetR expression cassette (P_hCMV_-PDD-TetR-pA_bGH_)  PDD expression cassette was PCR-amplified from pSG096 with oligos oSG029-oSG253, digested with EcoRI/SpeI and inserted into pTS2365 restricted with EcoRI/SpeI. | This work |
| pSG241 | Vector coding for constitutive PDD^(R53C, E78D, L88M)^-TetR.  (P_hCMV_-PDD^(R53C, E78D, L88M)^-TetR-pA_bGH_)  The mutant PDD was obtained from error-prone mutagenesis PCR on pSG240 using oligos oSG029-oSG253, digested with EcoRI/SpeI and inserted into pSG240 restricted with EcoRI/SpeI. | This work |
| pSG242 | Vector coding for constitutive PDD^(F80L, L88M)^-TetR.  (P_hCMV_-PDD^(F80L, L88M)^-TetR-pA_bGH_).  The mutant PDD was obtained from error-prone mutagenesis PCR on pSG240 using oligos oSG029-oSG253, digested with EcoRI/SpeI and inserted into pSG240 restricted with EcoRI/SpeI. | This work |
| pSG243 | Vector coding for constitutive PDD^(R53C, E78D, L88M)^-VPR.  (P_hCMV_-NLS-PDD^(R53C, E78D, L88M)^-VPR-pA_bGH_).  PDD^(R53C, E78D, L88M)^ was isolated from pSG241 by restriction digestion with EcoRI/SpeI and inserted into pSG138 restricted with EcoRI/SpeI. | This work |
| pSG244 | Vector coding for constitutive PDD^(F80L, L88M)^-VPR.  (P_hCMV_-NLS-PDD^(F80L, L88M)^-VPR-pA_bGH_).  PDD^(F80L, L88M)^ was isolated from pSG242 by restriction digestion with EcoRI/SpeI and inserted into pSG138 restricted with EcoRI/SpeI. | This work |
| pSG246 | Vector coding for cytosolically expressed PDD^m^-NLuc^N^.  (P_hCMV_-PDD^m^-NLuc^N^-pA_bGH_)  The construct was cloned by Gibson assembly. NLuc^N^ was PCR-amplified from pSG230 with oligos oSG238-oSG248; the backbone was PCR-amplified with oligos oSG247-oSG250 from pSG241. | This work |
| pSG247 | Vector coding for a cytosolically expressed PDD^m^-NLuc^C^. (P_hCMV_-PDD^m^-NLuc^C^-pA_bGH_)  NLuc^C^ was obtained by oligos dimerization of oSG239-oSG240, ligated into pSG241 PCR-amplified with oligos oSG063-oSG251 and restricted with NheI/HindIII. | This work |
| pSG250 | Vector coding for PDD-VPR-p2A.  (P_hCMV_-NLS-PDD-VPR-p2A-pA_bGH_)  The construct was cloned by Gibson assembly. PDD-VPR was PCR-amplified from pSG138 with oligos oSG029-oSG259; the backbone was amplified from pTS1214 with oligos oSG007-oSG178. | This work |
| pSG251 | Vector coding for PDD-VPR-p2A-PDD^m^-TetR.  (P_hCMV_-NLS-PDD-VPR-p2A-PDD^m^-TetR-pA_bGH_)  The construct was cloned by Gibson assembly. PDD^m^-TetR was PCR-amplified from pSG241 with oligos oSG053-oSG260; the backbone was amplified from pSG250 with oligos oSG007-oSG261. | This work |
| pSG256 | Vector coding for a constitutive NLS-rPDD-VPR.  (P_hCMV_-NLS-rPDD-VPR-pA_bGH_)  NLS-rPDD was isolated from a gene fragment gSG027 by restriction digestion with EcoRI/SpeI and ligated in pSG138 restricted with EcoRI/SpeI. | This work |
| pSG257 | Vector coding for a constitutive rPDD-TetR expression cassette (P_hCMV_-rPDD-TetR-pA_bGH_)  rPDD expression cassette was isolated from a gene fragment by restriction digestion with EcoRI/SpeI and ligated in pSG240 restricted with EcoRI/SpeI. | This work |
| pSG274 | Vector coding for a constitutive PAL.  (P_hCMV_-HA-PAL-pA_bGH_)  HA-PAL expression cassette was isolated from the gene fragment gSG029 by restriction digestion with EcoRI/HindIII and ligated into pTS1022 restricted with EcoRI/HindIII. | This work |
| pSG275 | Vector coding for a constitutive hIL4I1.  (P_hCMV_-hIL4I1-pA_bGH_)  hIL4I1 expression cassette was isolated from the gene fragment gSG030 by restriction digestion with EcoRI/NheI and ligated into pTS1022 restricted with EcoRI/NheI. | This work |
| pSG276 | Vector coding for a constitutive mIL4I1.  (P_hCMV_-mIL4I1-pA_bGH_)  The plasmid was cloned by Gibson assembly. mIL4I1expression cassette was PCR-amplified from the gene fragments gSG031 with oligos oSG029-oSG269 and gSG032 with oligos oSG053-oSG268 and cloned into pTS1022 amplified with oSG007-oSG063. | This work |
| pSG277 | Vector coding for a TetR-inducible PAL.  (O_TetO7_-P_min_-HA-PAL-pA_bGH_)    HA-PAL was isolated from the gene fragment gSG029 by restriction digestion with EcoRI/HindIII and ligated into pTS1017 restricted with EcoRI/HindIII. | This work |
| pSG279 | Vector coding for a TetR-inducible mIL4I1.  (O_TetO7_-P_min_-mIL4I1-pA_bGH_)  The vector was cloned by Gibson assembly. gSG031 and gSG032 were PCR-amplified with oligos oSG029-oSG269 and oSG053-oSG268 and cloned into pTS1017 PCR-amplified with oligos oSG007-oSG063. | This work |
| pSG280 | Vector coding for constitutive NLS-PDD-VP16.  (P_hCMV_-NLS-PDD-VP16-pA_bGH_)  NLS-PDD was isolated from pSG138 by restriction digestion with EcoRI/SpeI and ligated into pTS2367 restricted with EcoRI/SpeI. | This work |
| pSG281 | Vector coding for constitutive NLS-PDD-VP64.  (P_hCMV_-NLS-PDD-VP64-pA_bGH_)  NLS-PDD was isolated from pSG138 by restriction digestion with EcoRI/SpeI and ligated into pTS2368 restricted with EcoRI/SpeI. | This work |
| pSG284 | Vector coding for constitutive expression of PDD fused to a split TEV protease and localized in the ER.  P_hCMV_-ss-PDD-ssTEVp_1-118_-KDEL-pA_bGH_)  The vector was cloned by Gibson assembly. PDD was PCR-amplified from pSG138 with oligos oSG272-oSG273 and inserted into pMMH26 PCR-amplified with oligos oSG214-oSG215. | This work |
| pSG285 | Vector coding for constitutive expression of PDD fused to a split TEV protease and localized in the ER.  (P_hCMV_-ss-PDD-ssTEVp_119-245_-KDEL-pA_bGH_)  The vector was cloned by Gibson assembly. PDD was PCR-amplified from pSG138 with oligos oSG272-oSG274 and inserted into pMMH27 PCR-amplified with oligos oSG214-oSG218. | This work |
| pSG286 | Vector coding for constitutive expression of PPD^m^ fused to a split TEV protease and localized in the ER.  P_hCMV_-ss-PPD^m^-ssTEVp_1-118_-KDEL-pA_bGH_)  The vector was cloned by Gibson assembly. PPD^m^ was PCR-amplified from pSG241 with oligos oSG272-oSG273 and inserted into pMMH26 PCR-amplified with oligos oSG214-oSG215. | This work |
| pSG287 | Vector coding for constitutive expression of PPD^m^ fused to a split TEV protease and localized in the ER.  (P_hCMV_-SS-PPD^m^-ssTEVp_119-245_-KDEL-pA_bGH_)  The vector was cloned by Gibson assembly. PPD^m^ was PCR-amplified from pSG241 with oligos oSG272-oSG274 and inserted into pMMH27 PCR-amplified with oligos oSG214-oSG218. | This work |
| pSG302 | Vector coding for constitutive expression of PPD on the membrane.  (P_SV40_-ss-PDD-EpoR_m_-IL6RB_m_-p_AbGH_)  PDD was PCR-amplified from pSG138 with oligos oSG296-oSG297, digested with BamHI/EcoRI and ligated into pLeo619 digested with BamHI/EcoRI. | This work |
| pSG303 | Vector coding for constitutive expression of PPD^m^ on the membrane.  (P_SV40_-ss-PDD^m^-EpoR_m_-IL6RB_m_-p_AbGH_)  PDD^m^ was PCR-amplified from pSG214 with oligos oSG296-oSG297, digested with BamHI/EcoRI and ligated into pLeo619 digested with BamHI/EcoRI. | This work |
| pSG304 | Vector coding for constitutive secretion of PDD-NLuc^N^.  (P_hCMV_-ss-PDD-NLuc^N^)  The construct was cloned by PCR amplification of pSG230 with oligos oSG298-299. | This work |
| pSG305 | Vector coding for constitutive expression of PDD-NLuc^C^. (P_hCMV_-ss-PDD-NLuc^C^)  The construct was cloned by PCR amplification of pSG231 with oligos oSG298-oSG299. | This work |
| pSG310 | Vector coding for constitutive secretion of PDD^m^-NLuc^N^.  (P_hCMV_-ss-PDD^m^-NLuc^N^)  The construct was cloned by PCR amplification of pSG246 with oligos oSG298- oSG299. | This work |
| pSG311 | Vector coding for constitutive expression of PDD^m^-NLuc^C^. (P_hCMV_-ss-PDD^m^-NLuc^C^)  The construct was cloned by PCR amplification of pSG247 with oligos oSG298- oSG299. | This work |
| pSG316 | Vector coding for a TetR-inducible mIL4I1-p2A-SEAP.  (O_TetO7_-P_min_-mIL4I1-p2A-SEAP-pA_bGH_).  The plasmid was cloned by PCR amplification of pSG279 with oligos oSG063- oSG304. The amplified product was digested with AgeI/SbfI and ligated into pGU137 restricted with AgeI/SbfI. | This work |
| pSG328 | Vector coding for constitutive expression of PAL-p2A.  (P_hCMV_-PAL-p2A-pA_bGH_).  PAL was PCR-amplified from pSG274 with oligos oSG315-oSG316. The PCR product was digested with EcorI/SpeI and ligated into pTS1214 restricted with EcorI/SpeI. | This work |
| pSG329 | Vector coding for TetR-inducible PAL-p2A-mIL4I1-p2A-SEAP.  (O_TetO7_-P_min_-PAL-p2A-mIL4I1-p2A-SEAP-pA_bGH_)  PAL-p2A was isolated from pSG328 by restriction digestion with EcorI/NheI and ligated into pSG316 restricted with EcorI/SpeI. | This work |
| pSG342 | Tier3 vector coding for the Phe gene switch.  (5’ITR-cassette1-pA_bGH_::P_hCMV_-PDD-VPR-p2A-PDD_m_-TetR-pA_p36_::P_RPBSA_-YPet-p2A-PuroR-pA_p9_-3’ITR)  PDD-VPR-p2A-PDD_m_-TetR was isolated from pSG251 MluI/HindIII and ligated into pTS1029 restricted with BsaI. | This work |
| pSG344 | Tier3 vector coding for mIL4I1 expression under the control of the genetic switch.  (5’ITR-O_TetO7_-P_min_-mIL4I1-p2A-SEAP-pA_bGH_::P_hCMV_-PDD-VPR-p2A-PDD_m_-TetR-pA_p36_::P_RPBSA_-YPet-p2A-PuroR-pA_p9_-3’ITR)  O_TetO7_-P_min_-mIL4I1-p2A-SEAP was restricted from pSG316 with MluI/AgeI/SbfI and ligated into pSG342 restricted with MluI/SbfI. | This work |
| pSG346 | Tier3 vector coding for PAL and mIL4I1 expression under the control of the genetic switch.  (5’ITR-O_TetO7_-P_min_-PAL-p2A-mIL4I1-p2A-SEAP-pA_bGH_::P_hCMV_-PDD-VPR-p2A-PDD_m_-TetR-pA_p36_::P_RPBSA_-YPet-p2A-PuroR-pA_p9_-3’ITR)  O_TetO7_-P_min_-PAL-p2A-mIL4I1-p2A-SEAP was restricted from pSG329 with MluI/AgeI/SbfI and ligated into pSG342 restricted with MluI/SbfI. | This work |
| pSG372 | Tier3 vector coding for the Phe genetic switch.  (5’ITR-O_TetO7_-P_hCMV_-PDD-VPR-p2A-PDD_m_-TetR-pA_bGH_::P_RPBSA_-BlastR-p2A-mRuby-pA_p9_-3’ITR)  pSG251 was restricted with MluI/HindIII and ligated into pTS2344 restricted with MluI/HindIII. | This work |
| pSG408 | Vector coding for a nuclear-localized PDD^m^-p65.  (P_hCMV_-NLS-PDD^m^-p65-pA_bGH_)  PDD^m^-p65 was isolated from a gene fragment with EcoRI/HindIII and ligated into pTS1022 restricted with EcoRI/HindIII. | This work |
| pSG409 | Plasmid coding for a nuclear localized PDD-ZF10.  (P_hCMV_-NLS-PDD-ZF10-pA_bGH_)  PDD-ZF10 was isolated from a gene fragment with EcoRI/HindIII and ligated into pTS1022 restricted with EcoRI/HindIII. | This work |
| pSG410 | Plasmid coding for nuclear-localized ZF10-p65.  (P_hCMV_-NLS-ZF10-p65-pA_bGH_)  ZF10-p65 was isolated from a gene fragment with EcoRI/HindIII and ligated into pTS1022 restricted with EcoRI/HindIII. | This work |
| pSG411 | Reporter gene plasmid for ZF10 constructs.  (4*DBD_ZF10_-P_min_-SEAP-pA_bGH_)  4*DBD_ZF10_-P_min_ was isolated from a gene fragment with EcoRI/MluI and ligated into pTS1022 restricted with EcoRI/MluI. | This work |
| pSG414 | Vector coding for a nuclear localized PDD-p65.  (P_hCMV_-NLS-PDD-p65-pA_bGH_)  PDD-p65 was isolated from a gene fragment with EcoRI/HindIII and ligated into pTS1022 restricted with EcoRI/HindIII. | This work |
| pSG415 | Plasmid coding for a nuclear localized PDD^m^-ZF10.  (P_hCMV_-NLS-PDD^m^-ZF10-pA_bGH_)  PDD^m^-ZF10 was isolated from a gene fragment with EcoRI/HindIII and ligated into pTS1022 restricted with EcoRI/HindIII. | This work |
| pSG449 | Vector coding for constitutive secretion of XylR-NLuc^N^.  (P_hCMV_-ss-XylR-NLuc^N^)  The ss was added to pSG214 by PCR with oligos oSG299-oSG390. | This work |
| pSG450 | Vector coding for constitutive secretion of XylR-NLuc^C^.  (P_hCMV_-ss-XylR-NLuc^N^)  The ss was added to pSG215 by PCR with oligos oSG299-oSG390. | This work |
| pSG451 | Vector coding for nuclear-localized PDD^m^-GAL4DBD.  (P_hCMV_-NLS-PDD^m^-GAL4DBD-pA_bGH_)  GAL4DBD was isolated from pTS2373 with SpeI/HindIII and ligated into pSG243 restricted with SpeI/HindIII. | This work |
| pSG456 | Vector coding for constitutive HA-tagged mIL4I1.  (P_hCMV_-mIL4I1-GGGGS-HA-pA_bGH_)  GGGGS-HA was obtained by oligo dimerization of oSG393-oSG394 and inserted into pSG276 restricted with AgeI/HindIII. | This work |
| pSG457 | Vector coding for constitutive mIL4I1 with the natural secretion peptide replaced with ss.  (P_hCMV_-ss-mIL4i1^(22-630)^-GGGGS-HA-pA_bGH_)  mIL4i1^(22-630)^-GGGGS-HA was PCR-amplified from pSG456 with oligos oSG063-oSG395, restricted with SpeI/HindIII and ligated into pTS1012 restricted with SpeI/HindIII. | This work |
| pSG458 | Vector coding for HA-tagged mIL4i1^(1-561)^.  (P_hCMV_-mIL4i1^(1-561)^-GGGGS-HA-pA_bGH_)  GGGGS-HA was obtained by oligo dimerization of oSG393-oSG394 and inserted into pSG276 PCR-amplified with oligos oSG063-oSG396, and restricted with AgeI/HindIII | This work |
| pSG459 | Vector coding for HA-tagged mIL4i1^(1-509)^.  (P_hCMV_-mIL4i1^(1-509)^-GGGGS-HA-pA_bGH_)  GGGGS-HA was obtained by oligo dimerization of oSG393-oSG394 and inserted into pSG276 PCR-amplified with oligos oSG063-oSG397 and restricted with AgeI/HindIII | This work |
| pSG469 | Vector coding for a TetR-inducible PAL.  (O_TetO7_-P_min_-PAL-pA_bGH_)    HA-PAL was isolated from pSG328 by restriction digestion with EcoRI/SpeI and ligated into pTS1017 restricted with EcoRI/NheI. | This work |
| pSG494 | Tier3 vector coding for TetR-inducible mIL4I1.  (5’ITR-cassette1::O_TetO7_-P_min_-mIL4I1-p2A-SEAP-pA_p36_:: P_RPBSA_-YPet-p2A-PuroR-pA_p9_-3’ITR)  P_min_-mIL4I1-p2A-SEAP cassette was restricted from pSG316 with MluI/HindIII and ligated into pTS1029 digested with BsaI. | This work |
| pSG508 | Tier3 vector coding for TetR-inducible PAL and mIL4I1.  (5’ITR-O_TetO7_-P_min_-HA-PAL-pA_bGH_::O_TetO7_-P_min_-mIL4I1-p2A-SEAP-pA_p36_::P_RPBSA_-YPet-p2A-PuroR-pA_p9_-3’ITR)  O_TetO7_-P_min_-HA-PAL cassette was restricted from pSG277 with MluI/HindIII and ligated into pSG494 restricted with MluI/HindIII. | This work |
| pSG536 | Vector coding for constitutive PDD^(K14N)^-TetR.  (P_hCMV_-PDD^(K14N)^-TetR-pA_bGH_)  The mutant PDD was obtained from error-prone mutagenesis PCR on pSG240 using oligos oSG029-oSG253, digested with EcoRI/SpeI and inserted into pSG240 restricted with EcoRI/SpeI. | This work |
| pSG537 | Vector coding for constitutive PDD^(A4T, L54V)^-TetR.  (P_hCMV_-PDD^(A4T, L54V)^-TetR-pA_bGH_)  The mutant PDD was obtained from error-prone mutagenesis PCR on pSG240 using oligos oSG029-oSG253, digested with EcoRI/SpeI and inserted into pSG240 restricted with EcoRI/SpeI. | This work |
| pSG538 | Vector coding for constitutive PDD^(L198I)^-TetR.  (P_hCMV_-PDD^(L198I)^-TetR-pA_bGH_)  The mutant PDD was obtained from error-prone mutagenesis PCR on pSG240 using oligos oSG029-oSG253, digested with EcoRI/SpeI and inserted into pSG240 restricted with EcoRI/SpeI. | This work |
| pSG539 | Vector coding for constitutive PDD^(N32K)^-TetR.  (P_hCMV_-PDD^(N32K)^-TetR-pA_bGH_)  The mutant PDD was obtained from error-prone mutagenesis PCR on pSG240 using oligos oSG029-oSG253, digested with EcoRI/SpeI and inserted into pSG240 restricted with EcoRI/SpeI. | This work |
| pSG540 | Vector coding for constitutive PDD^(H107N)^-TetR.  (P_hCMV_-PDD^(H107N)^-TetR-pA_bGH_)  The mutant PDD was obtained from error-prone mutagenesis PCR on pSG240 using oligos oSG029-oSG253, digested with EcoRI/SpeI and inserted into pSG240 restricted with EcoRI/SpeI. | This work |

**Abbreviations and additional information**

**BlastR**, blasticidin resistance gene; **DBD_ZF10_**, ZF10 DNA binding domain from Li et. al., 2022; **EpoRm**, modified EpoR3A (F93A); **GAL4DBD,** DNA binding domain of GAL4 transcription factor; **HA,** hemagglutinin tag;  **hIL4I1,** human interleukin 4 induced 1; **IL6RB_m_,** mutant intracellular domain of IL-6 receptor subunit beta (Y759A); **KDEL,** lysine, aspartic acid, glutamic acid, leucine; **MCS**, multiple cloning site; **mIL4I1,** mouse interleukin 4 induced 1; **mRuby2**, red fluorescent protein; **NLS**, nuclear localization sequence; **NLuc**, NanoLuc luciferase; **NLuc^C^,** C-terminal part of split NLuc from Dixon et al., 2016; **NLuc^N^,** N-terminal part of split NLuc from Dixon et al., 2016; **O_TetO7_**, heptameric TetR-specific operator; **p2A**, 2A self-cleaving peptide; **hPAH**, human phenylalanine hydroxylase; p65, human transcription factor; **PAL**, phenylalanine ammonia lyase from *Photorhabdus luminescens*; **pA_bGH_**, polyadenylation signal from the bovine growth hormone; **pA_p36_** and **pA_p9_** synthetic polyadenylation signal; **pA_SV40_**, polyadenylation signal from simian virus 40; **PCR,** polymerase chain reaction; **P_hCMV_**, human cytomegalovirus immediate-early promoter; **PDD**, regulatory domain of the human PAH; **PDD_m_,** PDD^R53C, E78D, L88M^; **P_min_**, weak pGL4.23-derived minimal promoter; **P_RPBSA_,** synthetic constitutive promoter; **P5xUAS,** 5 repeats of GAL4 DNA binding sites from *Saccharomyces cerevisiae*; **PuroR**, puromycin resistance gene; **rPDD**, regulatory domain of the PAH from *Rattus norvegicus*; **SEAP**, human placental secreted alkaline phosphatase; **ss**, secretion signal; **ssTEVp**, split secretory tobacco etch virus protease; **TCS,** TEVp cleavage site; **TetR**, *E. coli* Tn10-derived tetracycline-dependent repressor; **TEV**, tobacco etch virus protease; **sTRSV,** hammerhead ribozyme engineered from the satellite RNA of the tobacco ringspot virus; **VHH_A52_**, variable domain of camelid heavy chain antibody against Reactive Red 120; **VP16**, herpes simplex virus-derived transactivation domain; **VP64**, four tandem repeats of VP16; **VPR***,* tripartite mammalian transactivator VP64-p65-Rta; **XylR**, xylose operon regulatory protein from *E. coli*; **YPet**, modified yellow fluorescent protein; **ZF10,** synthetic zinc finger from Li et. al., 2022.

Table S2 | Oligonucleotide sequences used in this work

| **Oligo ID** | **Oligonucleotide Sequence (5’-3’)** |
| --- | --- |
| oSG007 | catggtgaattccgcttcgaac |
| oSG012 | gacgtctcactggttccactgcggtcctggaaaa |
| oSG013 | cgtctctagaaagctttgaggccggc |
| oSG022 | gacgtctcactggttccactgcggtcctggaaaa |
| oSG023 | cgtctctttctagtactttattttctggaggg |
| oSG029 | tcgaagcggaattcaccatg |
| oSG032 | ctttcctctttttctttggcatggtgaattccgcttcgaa |
| oSG053 | gccggcctcaaagctttcta |
| oSG063 | tagaaagctttgaggccggc |
| oSG098 | tcgaagcggaattcaccatgtccactgcggtcctggaa |
| oSG099 | cactgtgtctttcttcttatctcgt |
| oSG101 | tcgaagcggaattcaccatgggacaggaaacaagctatat |
| oSG106 | cactgtgtctttcttcttatctcgt |
| oSG166 | gaaaaagaggaaagtctccactgcggtcctggaaaa |
| oSG167 | gaaaaagaggaaagtcggacaggaaacaagctatattg |
| oSG178 | ggtggttctggtggaagcg |
| oSG179 | ggaattcaccatgactagttccactgcggtcctggaa |
| oSG214 | ggcggaggtggttctggcg |
| oSG215 | ggcggaggtggttctggcg |
| oSG218 | aaatccatgagctccatggtctccg |
| oSG238 | aaagctttctatgagttgatggttactcgga |
| oSG239 | ctagtgtcacgggttatcgccttttcgaggagattttgtagaa |
| oSG240 | agctttctacaaaatctcctcgaaaaggcgataacccgtgaca |
| oSG246 | tcgagtgtgaagacactagcgagcatcacctctgagttca |
| oSG247 | atcaactcatagaaagctttgaggccggc |
| oSG248 | gctagtgtcttcacactcgaagattt |
| oSG250 | tcgagtgtgaagacactagccactgtgtctttcttcttatctcgt |
| oSG251 | agctagccactgtgtctttcttcttatctcgt |
| oSG253 | gacactagtcactgtgtctttcttcttatctcgt |
| oSG254 | gatgttcccgactacgccgggtcgtcttccactgcggtcctggaaaa |
| oSG256 | gtagtcgggaacatcgtatgggtacatggtgaattccgcttcga |
| oSG259 | ttccaccagaaccaccactagtcagagatgtgtcgaagatgg |
| oSG260 | tccactgcggtcctggaaaa |
| oSG261 | ttttccaggaccgcagtggaaggtccagggttctcctc |
| oSG268 | ccgatgttgttcttctcacc |
| oSG269 | ggtgagaagaacaacatcgg |
| oSG272 | tcctcggcctggccgtcgcctccactgcggtcctggaaaa |
| oSG273 | ccgccagaaccacctccgcccactgtgtctttcttcttatctcgt |
| oSG274 | accatggagctcatggatttcactgtgtctttcttcttatctcgt |
| oSG296 | gacggatcctccactgcggtcctggaaaa |
| oSG297 | tgcgaattctccggacactgtgtctttcttcttatctcgt |
| oSG298 | gggtactgctgctctgggttccaggttccactggttccactgcggtcctggaaaa |
| oSG299 | cccagagcagcagtacccatagcaggagtgtgtctgtctccatggtgaattccgcttcga |
| oSG304 | gtgaccggttgaatgatcgcctactctatgcatttg |
| oSG315 | cggaattcaccatgaaagcgaaggatgtgcagcc |
| oSG316 | ccactagtttcctcaagcattatctccggc |
| oSG390 | atgggtactgctgctctgggttccaggttccactggtttcactaagagacaccgcat |
| oSG393 | ccggtggcggtggtggctcatacccatacgatgttcccgactacgcctagaa |
| oSG394 | agctttctaggcgtagtcgggaacatcgtatgggtatgagccaccaccgcca |
| oSG395 | gtactagtgactggaaagcggccagc |
| oSG396 | ccaccggtggatccgctagcacttgtttcgacaagaagatgttta |
| oSG397 | ccaccggtggatccgctagcaccataattgttgttgattctaacgg |

**Table S3 | Codon-optimized gene fragments used in this work**

| **ID** | **Gene fragment sequence (5’-3’)** |
| --- | --- |
| gSG027 | gctgttcgaagcggaattcaccatgccaaagaaaaagaggaaagtcgcggcagttgtccttgagaacggagttctgagtaggaaactctctgactttggacaggaaacttcctacattgaagacaatagcaatcagaacggagcgatcagccttattttttccctgaaggaagaggtaggggcgctcgccaaggtgctccggctgtttgaggaaaacgacataaatcttacacacattgaatctcggccaagccgccttaacaaggatgaatatgagttttttacgtatctcgataaacgatcaaagcccgtactcggatctataattaagtcactccgaaatgacattggggccactgtccacgagctcagccgggataaagagaaaaatacagttactagtggttccggacgg |
| gSG029 | tcgaagcggaattcaccatgtacccatacgatgttcccgactacgccgggtcgtctactagtaaagcgaaggatgtgcagccgacaataatcataaataaaaatgggttgatctctcttgaggacatatatgatatagctataaaacagaagaaagtggaaatctctgctgaaattacagagttgctgacccatgggcgagagaagctggaagaaaaactgaactcaggtgaggtcatatatgggatcaacactggctttggcgggaacgccaatcttgtggttccgttcgaaaagatcagtgagcaccagcaaaatctgcttactttcttgtccgctggaactggtgactacatgtctaaaccgtgcattaaggcgagtcaattcacaattcttctgtctgtatgcaagggctggagcgccactaggcctattgttgcacaggctatagttgaccacattaacaatgacatagtgccgcttgtcccaaggtatggttccgtaggggcttctggcgatctcattcccttgagttatattgcccgggctttgtgtggcattgggaaggtgtactacatgggggctgaaatcgacgcagcagaagcaataaaacgggctgggctgacgcccctcagccttgaagcaaaggaggggctcgctttgattaatggaaccagagtcatgagcggtatatcagctatcacggtcataaagctggaaaaactctttaaggcgagcattagcgccatagcactcgcagtggaggcgctgctcgcatcccatgagcattacgacgcacgaatacaacaggttaaaaatcatccgggtcaaaaagcagtggcatccgcgctgaggaatcttctggcgggttccacacaaataaatctgttgactggtatcaaggaacaggcgaacaaagcatgtaggcatcaggaagttactcgcttgaatgatactttgcaagaagtatattcaatccgatgtgccccacagatcttggggatcgtacccgaatcactcgccacggcgaggaaaatattggagagagaagttataagcgctaacgacaatcccctcatcgacccagaaaatggagatgtactgcacggggggaattttatggggcaatatgttgcccgaactatggatgcccttaagctggacatcgcgctgatagcaaatcacctccacgctatagtagcgctcatgatggacaaccgattttcacgcggactgcccaactctttgagtccgactccgggtatgtaccaaggatttaaaggagtacagttgtcacaaaccgccctggtagctgcaatacgccacgattgcgctgcctcaggaatccatacactggcaaccgagcagtacaaccaagatatcgtcagtctcggtcttcacgcggcccaggatgttctggaaatggaacaaaaactgaggaatattgtcgctatgactatcttggtggtatgtcaagctatacatctgaggggaaatataagtgaaatcgcacccgaaacggcaaagttttaccatgccgttcgagaaatctcatcccctcttataacggaccgcgccctggacgaggatatcactagaattgctgacgctataatcaacgatcagttgcctttgccggagataatgcttgaggaagctagcggatccaccggtgtctagaaagctttg |
| gSG030 | agcggaattcaccatgactagtcccaacgatgacttctgtcctgggctaaccataaaggccatgggtgctgagagagccccccagaggcagccatgcaccctgcacctcctcgtcctcgtccccatcctcctcagcctggtggcctcccaggactggaaggctgaacgcagccaagaccccttcgagaaatgcatgcaggatcctgactatgagcagctgctcaaggtggtgacctgggggctcaatcggaccctgaagccccagagggtgattgtggttggcgctggtgtggccgggctggtggccgccaaggtgctcagcgatgctggacacaaggtcaccatcctggaggcagataacaggatcgggggccgcatcttcacctaccgggaccagaacatgggctggattggggagctgggagccatgcgcatgcccagctctcacaggatcctccacaagctctgccagggcctggggctcaacctgaccaagttcacccagtacgacaagaacacgtggacggaggtgcacgaagtgaagctgcgcaactatgtggtggagaaggtgcccgagaagctgggctacgccttgcgtccccaggaaaagggccactcgcccgaagacatctaccagatggctctcaaccaggccctcaaagacctcaaggcactgggctgcagaaaggcgatgaagaagtttgaaaggcacacgctcttggaatatcttctcggggaggggaacctgagccggccggccgtgcagcttctgggagacgtgatgtccgaggatggcttcttctatctcagcttcgccgaggccctccgggcccacagctgcctcagcgacagactccagtacagccgcatcgtgggtggctgggacctgctgccgcgcgcgctgctgagctcgctgtccgggcttgtgctgttgaacgcgcccgtggtggcgatgacccagggaccgcacgatgtgcacgtgcagatcgagacctctcccccggcgcggaatctgaaggtgctgaaggccgacgtggtgctgctgacggcgagcggaccggcggtgaagcgcatcaccttctcgccgccgctgccccgccacatgcaggaggcgctgcggaggctgcactacgtgccggccaccaaggtgttcctatcgttccgcaggcccttctggcgcgaggagcacattgaaggcggccactcaaacaccgatcgcccgtcgcgcatgattttctacccgccgccgcgcgagggcgcgctgctgctggcctcgtacacgtggtcggacgcggcggcagcgttcgccggcttgagccgggaagaggcgttgcgcttggcgctcgacgacgtggcggcattgcacgggcctgtcgtgcgccagctctgggacggcaccggcgtcgtcaagcgttgggcggaggaccagcacagccagggtggctttgtggtacagccgccggcgctctggcaaaccgaaaaggatgactggacggtcccttatggccgcatctactttgccggcgagcacaccgcctacccgcacggctgggtggagacggcggtcaagtcggcgctgcgcgccgccatcaagatcaacagccggaaggggcctgcatcggacacggccagccccgaggggcacgcatctgacatggaggggcaggggcatgtgcatggggtggccagcagcccctcgcatgacctggcaaaggaagaaggcagccaccctccagtccaaggccagttatctctccaaaacacgacccacacgaggacctcgcatgctagcggatcca |
| gSG031 | gagacccaagctgttcgaagcggaattcaccatgactagtatggctggcttggctctgcgcctggtactcgcagcgacgttgctcgggctggctggaagcctcgactggaaagcggccagctcattgaatccaatcgagaaatgtatggaggatcatgattacgaacagctcctcaaggttgtaacgctcgggcttaacagaaccagtaaaccccaaaaggtggtagttgtcggggcaggggtagcaggattggtcgcagctaaaatgctttctgatgctggtcacaaagtgacgatattggaggcggacaatcgaatcggtggtcggatttttacgtttcgcgatgagaaaacgggatggatcggagaactgggcgcaatgagaatgccctcctctcatagaatactgcataaattgtgccggacactgggccttaatcttactcagtttacacagtatgatgaaaatacctggacagaggtccataacgtcaagctcagaaattacgttgttgaaaagatgccagaaaagttgggctacaatctgaataatcgcgaacgcggccacagccccgaagacatttatcaaatggcactgaataaagccttcaaagacttgaaggcacttggctgcaaaaaagcgatgaacaaattcaataagcataccctccttgagtatctccttgaggaaggcaatctctctcgaccggcagtccaactgcttggcgacgtcatgtctgaagagggcttcttctaccttagctttgctgaagctctgcgggcccacgcatgcctttctgaccggctgcggtattctcgcatagtggggggttgggaccttctgccccgagcgcttcttagttccttgtctggagccctgcttcttaatgctcccgtcgtctccatcacacaagggcggaatgatgtaagggttcatattgctacaagtctccatagcgaaaaaacgcttactgccgatgttgttcttctcaccgcatctgggccagcgcttcagaggattacattttcccccccgttgactcgcaaacgacaggaagccttgcgagctctccattatgtagcggccagcaaggtctttctttcatttaggaggcccttctggcatgaagagcatatagagggcggtcatagtaacacagatcgaccatcccgcctgattttttaccctgcgcgaggtgaagggagtctgctccttgcctcctacacctggagtgatgcggccgcacccttcgccggactgtccacggaccagactctgcgccttgtcctgcaagatgtagcggcactgcatgggcccgtggtgttccgactctgggacggacggggtgtcgtcaaacggtgggcagaagatccgcattcacagggaggctttgtagtccaaccgcctctctatggaagagaggcggaagactatgactggtcagcccccttcggtcgaatctacttcgcgggagaacatacagcattgcctcacgggtgggtcgagacagctgttaaatcaggccttcgcgcagccgttagaatcaacaacaattatggttacggggaggtcgatccacaaatgatggagcatgcctacgccgaggctaactatctcgaccagtatcccgaaggagagagaccggaggagcagcaggcccgcgaagaagtatcaccagacgagcaagagccaagtcataaacatcttcttgtcgaaacaagtccagaggggcaacagcacgcatttgttgaagcgatcccggaactgcaaggacacgtattcgtagagactgtcccgcaagaaaagggccacgcgcatcaaaacatatatccctccgagcacgttcaagtccatggtgaagttatccctgagtggcatggtcacggcggatctggcacgccacaaatgcatagagtaggcgatcattcagctagcggatccaccggtgtctagaaagctttgaggccggcctgcagggatcagcctcgac |
| gSG032 | gagacccaagctgttcgaagcggaattcaccatgactagtatggctggcttggctctgcgcctggtactcgcagcgacgttgctcgggctggctggaagcctcgactggaaagcggccagctcattgaatccaatcgagaaatgtatggaggatcatgattacgaacagctcctcaaggttgtaacgctcgggcttaacagaaccagtaaaccccaaaaggtggtagttgtcggggcaggggtagcaggattggtcgcagctaaaatgctttctgatgctggtcacaaagtgacgatattggaggcggacaatcgaatcggtggtcggatttttacgtttcgcgatgagaaaacgggatggatcggagaactgggcgcaatgagaatgccctcctctcatagaatactgcataaattgtgccggacactgggccttaatcttactcagtttacacagtatgatgaaaatacctggacagaggtccataacgtcaagctcagaaattacgttgttgaaaagatgccagaaaagttgggctacaatctgaataatcgcgaacgcggccacagccccgaagacatttatcaaatggcactgaataaagccttcaaagacttgaaggcacttggctgcaaaaaagcgatgaacaaattcaataagcataccctccttgagtatctccttgaggaaggcaatctctctcgaccggcagtccaactgcttggcgacgtcatgtctgaagagggcttcttctaccttagctttgctgaagctctgcgggcccacgcatgcctttctgaccggctgcggtattctcgcatagtggggggttgggaccttctgccccgagcgcttcttagttccttgtctggagccctgcttcttaatgctcccgtcgtctccatcacacaagggcggaatgatgtaagggttcatattgctacaagtctccatagcgaaaaaacgcttactgccgatgttgttcttctcaccgcatctgggccagcgcttcagaggattacattttcccccccgttgactcgcaaacgacaggaagccttgcgagctctccattatgtagcggccagcaaggtctttctttcatttaggaggcccttctggcatgaagagcatatagagggcggtcatagtaacacagatcgaccatcccgcctgattttttaccctgcgcgaggtgaagggagtctgctccttgcctcctacacctggagtgatgcggccgcacccttcgccggactgtccacggaccagactctgcgccttgtcctgcaagatgtagcggcactgcatgggcccgtggtgttccgactctgggacggacggggtgtcgtcaaacggtgggcagaagatccgcattcacagggaggctttgtagtccaaccgcctctctatggaagagaggcggaagactatgactggtcagcccccttcggtcgaatctacttcgcgggagaacatacagcattgcctcacgggtgggtcgagacagctgttaaatcaggccttcgcgcagccgttagaatcaacaacaattatggttacggggaggtcgatccacaaatgatggagcatgcctacgccgaggctaactatctcgaccagtatcccgaaggagagagaccggaggagcagcaggcccgcgaagaagtatcaccagacgagcaagagccaagtcataaacatcttcttgtcgaaacaagtccagaggggcaacagcacgcatttgttgaagcgatcccggaactgcaaggacacgtattcgtagagactgtcccgcaagaaaagggccacgcgcatcaaaacatatatccctccgagcacgttcaagtccatggtgaagttatccctgagtggcatggtcacggcggatctggcacgccacaaatgcatagagtaggcgatcattcagctagcggatccaccggtgtctagaaagctttgaggccggcctgcagggatcagcctcgac |

**Table S4 | Plasmids and details for transient transfection experiments**

The amount of plasmid transfected per well of a 96-well plate is shown. From top to bottom represents from left to right in the figure.

| Figure 1b | 30 ng pTS1017, 60 ng pSG240, 60 ng pSG280  30 ng pTS1017, 60 ng pSG240, 60 ng pSG281  30 ng pTS1017, 60 ng pSG240, 60 ng pSG138 |
| --- | --- |
| Figure 1c | 30 ng pTS1017, 120 ng Filler plasmid  30 ng pTS1017, 60 ng pSG138, 60 ng Filler plasmid  30 ng pTS1017, 60 ng pSG240, 60 ng Filler plasmid  30 ng pTS1017, 60 ng pSG138, 60 ng pSG240 |
| Figure 1d | 30 ng pTS1017, 60 ng pSG138, 60 ng pSG240  Cells were grown in normal DMEM before exposure to an excess of the single aromatic amino acids. |
| Figure 1e | 30 ng pTS1017, 60 ng pSG138, 60 ng pSG240 |
| Figure 2a | 50 ng pSG409, 50 ng pSG411, 50 ng pSG414 |
| Figure 2b | 30 ng pAna225, 60 ng pSG153, 60 ng pSG138 |
| Figure 2c | 30 ng pSG230, 30 ng pSG231, 60 ng Filler  30 ng pSG214, 30 ng pSG215, 60 ng Filler |
| Figure 2d | 30 ng pSG304, 30 ng pSG305, 90 ng Filler  30 ng pSG449, 30 ng pSG450, 90 ng Filler |
| Figure 2e | 5 ng pSG284, 5 ng pSG285, 5 ng pMMH91, 105 ng Filler  5 ng pMMH26, 5 ng pMMH27, 5 ng pMMH91, 105 ng Filler  5 ng pMMH91, 115 ng Filler |
| Figure 2f | 100 ng pSG302, 25 ng pLS13, 5 ng pLS15  25 ng pLS13, 5 ng pLS15  100 ng pLeo619, 25 ng pLS13, 5 ng pLS15  25 ng pLS13, 5 ng pLS15 |
| Figure 3a | 30 ng pSG100, 60 ng pSG240, 60 ng pSG234  30 ng pSG100, 60 ng pSG240, 60 ng pSG235  30 ng pSG100, 60 ng pSG240, 60 ng pSG236  30 ng pSG100, 60 ng pSG138, 60 ng pSG241  30 ng pSG100, 60 ng pSG138, 60 ng pSG242  30 ng pSG100, 60 ng pSG240, 60 ng pSG138 |
| Figure 3b | 30 ng pSG100, 60 ng pSG241, 60 ng pSG138  30 ng pSG100, 60 ng pSG240, 60 ng pSG243  30 ng pSG100, 60 ng pSG241, 60 ng pSG243  30 ng pSG100, 60 ng pSG240, 60 ng pSG138 |
| Figure 3c | 30 ng pSG100, 60 ng pSG138, 60 ng pSG241 |
| Figure 3d | 30 ng pSG100, 60 ng pSG138, 60 ng pSG240  30 ng pSG100, 60 ng pSG138, 60 ng pSG241 |
| Figure 4b | 75 ng pSG274, 75 ng Filler  75 ng pSG276, 75 ng Filler  75 ng pSG274, 75 ng pSG276  150 ng pFox12 |
| Figure 4d | 30 ng pSG100, 90 ng Filler |
| Figure S1a | 5 ng pTS1022, 95 ng Filler |
| Figure S1b | 30 ng pTS1017, 60 ng pSG096, 60 ng pSG240  30 ng pTS1017, 60 ng pSG138, 60 ng pSG240  30 ng pTS1017, 60 ng pSG240, 60 ng Filler |
| Figure S1c | 30 ng pTS1017, 60 ng pSG256, 60 ng pSG257  30 ng pTS1017, 60 ng pSG138, 60 ng pSG240 |
| Figure S1d | 30 ng pTS1017, 60 ng pSG140, 60 ng pSG199  30 ng pTS1017, 60 ng pSG138, 60 ng pSG240 |
| Figure S1e | 50 ng pSG410, 50 ng pSG411, 50 ng Filler  50 ng pSG409, 50 ng pSG411, 50 ng Filler  50 ng pSG411, 100 ng Filler |
| Figure S2a | 30 ng pSG100, 60 ng pSG240, 60 ng of each member of the PDD-VPR mutagenesis library  Control: 30 ng pSG100, 60 ng pSG240, 60 ng pSG138 |
| Figure S2b | 30 ng pSG100, 60 ng pSG138, 60 ng of each member of the PDD-TetR mutagenesis library  Control: 30 ng pSG100, 60 ng pSG240, 60 ng pSG138 |
| Figure S2c | 30 ng pSG100, 60 ng pSG240, 60 ng pSG234  30 ng pSG100, 60 ng pSG240, 60 ng pSG236  30 ng pSG100, 60 ng pSG240, 60 ng pSG235  30 ng pSG100, 60 ng pSG240, 60 ng pSG138 |
| Figure S2d | 30 ng pSG100, 60 ng pSG138, 60 ng pSG536  30 ng pSG100, 60 ng pSG138, 60 ng pSG242  30 ng pSG100, 60 ng pSG138, 60 ng pSG537  30 ng pSG100, 60 ng pSG138, 60 ng pSG538  30 ng pSG100, 60 ng pSG138, 60 ng pSG241  30 ng pSG100, 60 ng pSG138, 60 ng pSG539  30 ng pSG100, 60 ng pSG138, 60 ng pSG540  30 ng pSG100, 60 ng pSG240, 60 ng pSG138 |
| Figure S2e | 30 pSG100, 60 ng pSG138, 60 ng pSG241 |
| Figure S3a | 30 ng pTS1017, 60 ng pSG241, 60 ng pSG280  30 ng pTS1017, 60 ng pSG241, 60 ng pSG281  30 ng pTS1017, 60 ng pSG241, 60 ng pSG138 |
| Figure S3b | 50 ng pSG408, 50 ng pSG409, 50 ng pSG411  50 ng pSG414, 50 ng pSG415, 50 ng pSG411  50 ng pSG408, 50 ng pSG415, 50 ng pSG411 |
| Figure S3c | 30 ng pAna225, 60 ng pSG451, 60 ng pSG138  30 ng pAna225, 120 ng Filler |
| Figure S3d | 30 ng pSG246, 30 ng pSG231, 60 ng Filler  30 ng pSG230, 30 ng pSG247, 60 ng Filler  30 ng pSG246, 30 ng pSG247, 60 ng Filler |
| Figure S3e | 30 ng pSG310, 30 ng pSG305, 90 ng Filler  30 ng pSG304, 30 ng pSG311, 90 ng Filler  30 ng pSG310, 30 ng pSG311, 90 ng Filler |
| Figure S3f | 5 ng pSG286, 5 ng pSG285, 5 ng pMMH91, 105 ng Filler  5 ng pSG284, 5 ng pSG287, 5 ng pMMH91, 105 ng Filler  5 ng pSG286, 5 ng pSG287, 5 ng pMMH91, 105 ng Filler |
| Figure S3g | 100 ng pSG303, 25 ng pLS13, 5 ng pLS15  50 ng pSG302, 50 ng pSG303, 25 ng pLS13, 5 ng pLS15 |
| Figure S4a | 150 ng pSG233  150 ng pSG274  150 ng pSG276  150 ng pSG275  150 ng pFox12 |
| Figure S4b | 75 ng pSG274, 75 ng Filler  75 ng pSG276, 75 ng Filler  75 ng pSG274, 75 ng pSG276  150 ng pFox12 |
| Figure S4c | 500 ng pSG274  500 ng pSG456  500 ng pSG457  500 ng pSG458  500 ng pSG459  500 ng Filler  The experiment was carried out in 24-well plates. |
| Figure S4d | 600 ng pSG274  200 ng pSG138, 200 ng pSG241, 200 ng pSG469  600 ng pFox12  The experiment was carried out in 24-well plates. |
| Figure S4e | 600 ng pSG276  200 ng pSG138, 200 ng pSG241, 200 ng pSG279  600 ng pFox12  The experiment was carried out in 24-well plates. |
| Figure S5e | PRO cells transfected with 100s ng pSG100  The experiment was carried out in 24-well plates. |

**Supplementary References**

Catherine Y Seiler and others, DNASU plasmid and PSI:Biology-Materials repositories: resources to accelerate biological research, *Nucleic Acids Research*, **42**, D1253–D1260 (2014).

Dixon AS, Schwinn MK, Hall MP, Zimmerman K, Otto P, Lubben TH, Butler BL, Binkowski BF, MacHleidt T, Kirkland TA, Wood MG, Eggers CT, Encell LP, Wood K. NanoLuc Complementation Reporter Optimized for Accurate Measurement of Protein Interactions in Cells. *ACS Chem. Biol.* **11**, 400–408 (2016).

Franko N, Teixeira AP, Xue S, Charpin-El Hamri G, Fussenegger M. Design of modular autoproteolytic gene switches responsive to anti-coronavirus drug candidates. *Nat Commun* **12**, 6786 (2021).

Galvan S, Madderson O, Xue S, Teixeira AP, Fussenegger M. Regulation of Transgene Expression by the Natural Sweetener Xylose. *Adv. Sci*. **2203193** (2022).

Haellman V, Strittmatter T, Bertschi A, Stücheli P, Fussenegger M. A versatile plasmid architecture for mammalian synthetic biology (VAMSyB). *Metab. Eng*. **66**, 41– 50 (2021).

Li HS, Israni D V., Gagnon KA, Gan KA, Raymond MH, Sander JD, Roybal KT, Joung JK, Wong WW, Khalil AS. 2022. Multidimensional control of therapeutic human cell function with synthetic gene circuits. *Science* **378**, 1227–1234 (2022).

Mahameed M, Xue S, Stefanov BA, Charpin-El Hamri G, Fussenegger, M. Engineering a Rapid Insulin Release System Controlled By Oral Drug Administration. *Adv. Sci.*, **2105619** (2022).

Schukur L, Geering B, Charpin-El Hamri G, Fussenegger M. Implantable synthetic cytokine converter cells with AND-gate logic treat experimental psoriasis. *Sci. Transl. Med.* **7** (2015).

Scheller L, Strittmatter T, Fuchs D, Bojar D, Fussenegger M. Generalized extracellular molecule sensor platform for programming cellular behavior article. *Nat. Chem. Biol*. **14**, 723–729 (2018).
